# Identification of two odorant receptors tuned to alarm pheromone compounds in the honey bee *Apis mellifera*

**DOI:** 10.1038/s42003-025-09391-z

**Published:** 2025-12-23

**Authors:** Benjamin Andreu, Julia Mariette, Alizée Delarue, Virginie Larcher, Amandine Hueber, David Touboul, Nicolas Montagné, Thomas Chertemps, Emmanuelle Jacquin-Joly, Julie Carcaud, Jean-Christophe Sandoz

**Affiliations:** 1 Evolution, Genomes, Behavior and Ecology, Université Paris-Saclay (EGCE), CNRS, IRD, Gif-sur-Yvette, France; 2 Institut de Chimie des Substances Naturelles, UPR 2301, Université Paris-Saclay, CNRS, Gif-sur-Yvette, France; 3 Laboratoire de Chimie Moléculaire (LCM), CNRS, École polytechnique, Institut Polytechnique de Paris, Palaiseau, France; 4 Sorbonne Université, INRAE, CNRS, IRD, UPEC, Université Paris-Cité, Institute of Ecology and Environmental Sciences of Paris (iEES-Paris), Paris, France; 5 Institut universitaire de France (IUF), Paris, France

**Keywords:** Olfactory receptors, Social evolution

## Abstract

Being social insects, honey bees use an array of pheromones to facilitate intraspecific communication, ensuring colony cohesion in a wide range of contexts. The honey bee represents an attractive model for studying the neurobiological basis of pheromonal processing, given that their pheromones are well characterized and their olfactory pathway has been extensively studied. Despite substantial knowledge acquired on olfactory processing in this species, the mechanism of pheromonal coding remains poorly understood. In particular, olfactory receptors (ORs) detecting social pheromones are still unknown. In this study, we used heterologous expression in the Drosophila “empty neuron system”, coupled with transcuticular calcium imaging and electrophysiology. We deorphanized two odorant receptors, *Amel*OR136 and *Amel*OR109, which detect constituents of the alarm pheromone. *Amel*OR136 exhibits a sparse coding strategy, suggesting a finely tuned mechanism for efficient communication in alarm situations. In contrast, *Amel*OR109 is a more broadly-tuned receptor, responding to diverse odorants, including pheromones.

## Abstract (web summary)

Heterologous expression in the *Drosophila* empty neuron system, coupled with transcuticular calcium imaging and electrophysiology, allowed to deorphanize two odorant receptors of the honey bee which detect constituents of the alarm pheromone.

## Introduction

Sociality stands as an important evolutionary milestone, representing the transition from solitary life forms to group-oriented lifestyles^1^. Eusociality is the highest level of social organization, characterized by the division of individuals into castes with specialized roles, in particular regarding reproduction. Insects present the highest number of transitions towards eusociality, especially within the order of the Hymenoptera, with at least nine independent apparitions^2^. Eusocial insects exhibit remarkable cohesion and hierarchical organization, thanks to an elaborate communication between individuals, based in large part on the use of pheromones^3^. Social insects thus employ a diverse array of pheromones, in a multitude of contexts^4^. Moreover the chemical structures of these pheromones exhibit a remarkable diversity, encompassing variations in carbon chain length, functional group, and other characteristics^5^.

Among social insects, the honey bee *Apis mellifera* is known to use one of the largest panels of characterized pheromonal compounds, with over fifty identified compounds^3,6,7^. The queen emits a *queen mandibular pheromone* (QMP), which suppresses queen rearing, inhibits worker ovarian development, and also act as a sexual pheromone to attract males^8^. When confronted to a threat, worker bees release the *alarm pheromone*, which allows them to warn and attract other colony members and support colony defense^9–11^. The *alarm pheromone* is released by the Koschevnikov gland associated with the sting. Its major component, isopentyl acetate (IPA), triggers defensive and aggressive responses, including coordinated attacks^12^. Furthermore, workers also release an *aggregation pheromone*, which is employed in particular during swarm formation, signaling the group to gather and attract other workers towards important locations. Foragers that are successful at locating food, water and nesting sites use a *waggle dance pheromone* to recruit and stimulate other foragers to follow their recruitment dance and to search for these resources^13^. Finally, pheromone emitted by the brood (*brood pheromone*) mainly promotes brood care, inhibits the sexual maturation of worker bees and regulates the number of nurse bees and foragers^14–16^.

The large number of odorant cues used for communication in eusocial species, especially pheromones, has favored the emergence of an olfactory system capable of detecting and accurately processing these signals to produce appropriate behaviors. In insects, odorants are mainly detected at the level of the insect’s antennae^17^. These structures are covered with sensory hairs known as olfactory sensilla^18^, housing olfactory sensory neurons (OSNs)^17^. Odorant molecules penetrate the sensillum through its pores and reach the OSN dendrites either by diffusion into the hemolymph or through transport by odorant-binding proteins present in the lymph^19^. Upon reaching the OSN membrane, molecules bind to odorant receptors (ORs), which are seven-transmembrane proteins^20,21^ forming a heterodimer with a highly conserved co-receptor known as Orco^22^. Most OSNs express only one type of OR, and upon binding with an odorant, the OSNs depolarize and generate action potentials that are transmitted along their axons to the brain. Each insect species has evolved a repertoire of ORs that is tailored to its ecological needs^23–27^. The message generated by the OSNs is further relayed to the antennal lobe (AL), the primary olfactory center of the insect brain, where OSNs synapse with two other types of neurons, projection neurons and local interneurons, within spheroidal units called glomeruli^28,29^. After local processing, the message is conveyed to the higher order centers, the lateral horn and the mushroom bodies^30–32^.

This sophisticated nervous system allows the processing of pheromonal compounds, but how does it cope with the multiplication of such ecologically-relevant compounds found in social insects? One strategy for identifying how pheromonal compounds are detected and processed by the honey bee brain is to focus on the periphery of the olfactory system and to determine the response spectra of individual ORs. In honey bees, among the 170 ORs identified in the genome^33,34^, only three have been deorphanized (i.e. their ligand has been identified) and characterized thus far. Two receptors, *Amel*OR151 and *Amel*OR152 are known to detect floral compounds^35^, while *Amel*OR11 has been shown to detect 9-ODA, the major component of the QMP, involved in both sexual and social behaviors^36,37^. In this study, exploring a list of ORs chosen on the basis of available phylogenetic and transcriptomic data, we deorphanized two *A. mellifera* ORs belonging to the 9-exon clade, which strongly respond to honey bee alarm pheromone compounds, in particular the major component isopentyl acetate (IPA). One of these receptors appeared to be highly specific to alarm compounds, while the second displayed a more generalist response pattern. This work paves the way to the neurogenetic characterization of the role of these receptors in mediating bees’ behavioral response to alarm pheromones. Ultimately, it shall help understand how the social insect olfactory system makes sense of the plethora of social pheromones used for colony cohesion.

## Results

In this study, we aimed to identify honey bee ORs tuned to social pheromones. A preliminary screening of the responses of *Drosophila* OSNs expressing each of the 8 ORs to a range of honey bee extracts allowed to identify two candidate social pheromone receptors, *Amel*OR136 and *Amel*OR109. We first used the high throughput of transcuticular calcium imaging^36,38^ to systematically screen the responses of the OSNs expressing these receptors to a range of pheromonal blends and individual pheromonal compounds. In a second phase, single sensillum electrophysiological recordings were used to characterize these receptors’ tuning range and sensitivity. In the following, for simplicity, we refer to the responses of the receptors (for instance *AmelOR136*), although it is clear that the observed responses are the product of the whole OSN expressing the receptor.

### Responses to pheromonal blends

**Fig. 1:**
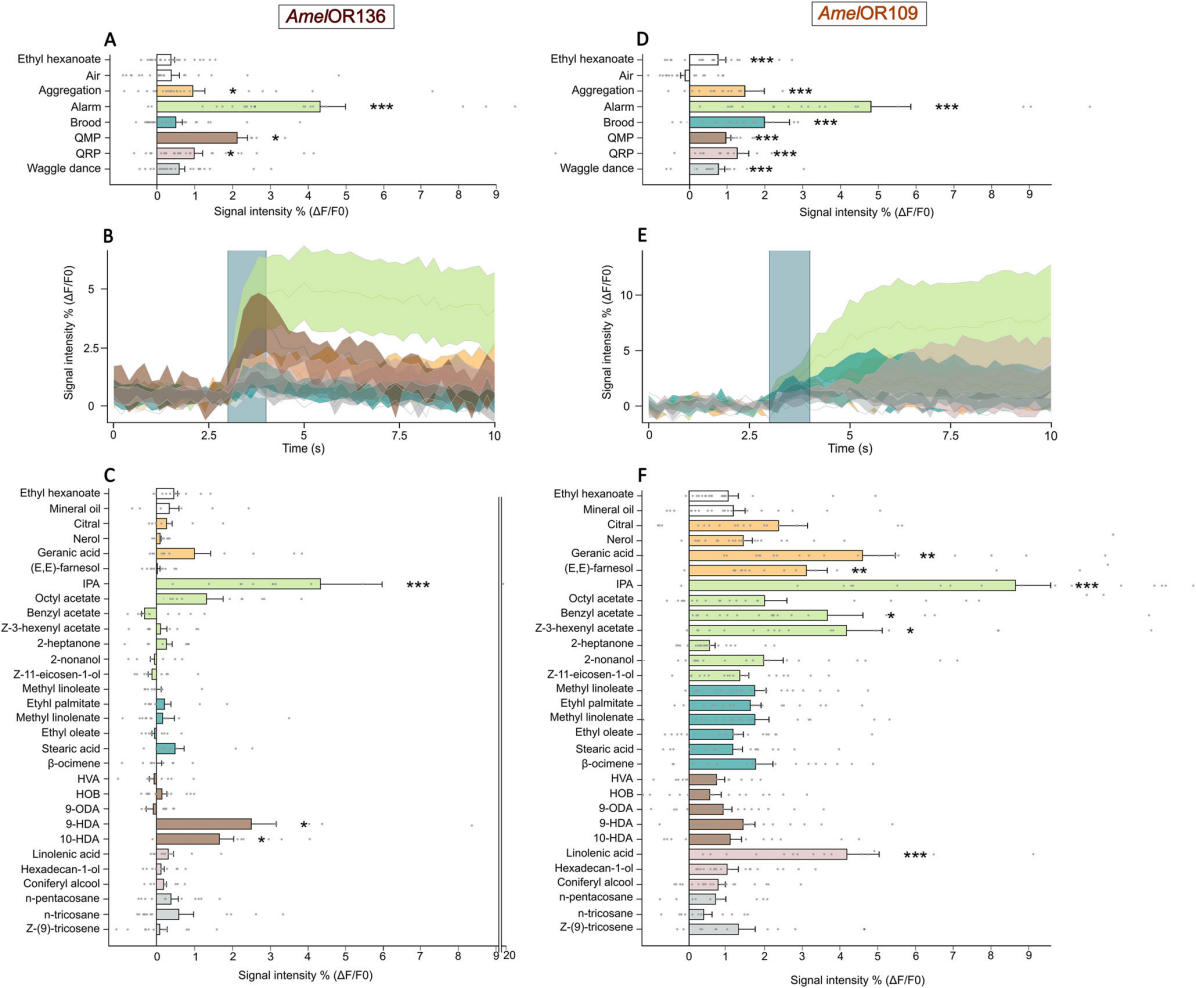
Responses of *Amel*OR136 and *Amel*OR109 to pheromonal blends and individual compounds recorded using calcium imaging (ΔF/F0). **A**, **D** Mean response intensity of *Amel*OR136 (**A**, *n* = 27 individuals) and *Amel*OR109 (**D**, *n* = 20 individuals) in response to the presentation of different pheromonal blends: aggregation pheromone (orange), alarm pheromone (green),  brood pheromone (blue), queen mandibular pheromone (QMP, brown), queen retinue pheromone (QRP, pink), and waggle dance pheromone (grey) blends. Alarm pheromone (***p < 0.001) and QMP, QRP and aggregation blends significantly activate *AmelOR*136 in comparison to the air control (*p < 0.01), whereas all blends activate *Amel*OR109 (***p < 0.001). **B**, **E** Time course of odor-evoked responses (average of 27 individuals for *Amel*OR136 and 20 individuals for *Amel*OR109) to the presentation of the 6 pheromonal blends (blue bar) for *Amel*OR136 **B** and for *Amel*OR109 **E** ± SEM. **C**, **F** Mean response of *Amel*OR136 (**C**, *n* = 11) and for *Amel*OR109 (**F**, *n* = 20) to the presentation of the individual pheromonal compounds tested at 1000 µg. Only isopentyl acetate (IPA), 9-hydroxy-(E)-2-decenoic acid (9-HDA) and 10-hydroxy-2-decenoic acid (10-HDA) significantly activate *Amel*OR136 (**C**, *p < 0.05), whereas *Amel*OR109 is activated by several odorants (**F**, *p < 0.05, **p < 0.01, ***p < 0.001).

The receptor *Amel*OR136 showed significant calcium response to several pheromonal blends in comparison to the air control (Fig. 1A; Friedman ANOVA, χ^2^ = 45.2, df = 6, p = 4.3 E-08). First, the presentation of the alarm pheromone blend triggered a robust and statistically significant response in comparison to the air control (Fig. 1A, Wilcoxon post hoc test, p = 7.8E-05). The calcium response persisted over time following stimulation, still exhibiting activity distinct from the baseline seven seconds after stimulation (Fig. 1B). The queen mandibular pheromone blend (QMP) also activated *Amel*OR136 (Fig. 1A, p = 0.04), as well as the aggregation and queen retinue (QRP) blends, albeit to a lesser extent (p = 0.043 in both cases). The responses to the other pheromonal blends (brood and waggle dance pheromones) were not different from the air control (Fig. 1A, all p-values > 0.05).

To identify the individual odorants that induced responses to the pheromone blends, all the compounds were then presented individually (Fig. 1C). The response of *Amel*OR136 to the alarm pheromone blend could be attributed to the sole presentation of isopentyl acetate (Friedman ANOVA, χ^2^ = 117.0, df = 29, p = 1.56 E-12, Wilcoxon post hoc test, *p* = 0.01). Concerning the QMP blend, *Amel*OR136 was activated by 9-HDA (*p* = 0.011), with a weaker response to 10-HDA (*p* = 0.01; Fig. 1C). No other compound induced any significant response of *Amel*OR136, suggesting that this odorant receptor is specific to a few odorants.

The receptor *Amel*OR109 also demonstrated significant responses to pheromonal blends compared to the air control (Fig. 1D; Friedman ANOVA, χ^2^ = 64.57, df = 7, *p* = 1.84 E-11). Remarkably, all the pheromonal blends triggered responses from *Amel*OR109 (Fig. 1D; Wilcoxon post hoc test, *p* < 0.003). It is noteworthy that the responses to these pheromonal blends varied, with the alarm pheromone eliciting the strongest response (Fig. 1D; *p* = 2.6E-05). In general, responses started slowly and persisted for more than seven seconds following stimulation (Fig. 1E). Presentation of the aggregation pheromone blend (Fig. 1D; *p* = 9.3E-05) elicited a response comparable to that obtained with the brood pheromone blend (Fig. 1D; *p* < 0.001). Similarly, both QMP and QRP blends induced a lower but significant response from *Amel*OR109 (Fig. 1D; QMP vs air: *p* = 1.3E-04, QRP vs air: *p* = 2.4E-04).

As above, the components of the different pheromonal blends were tested individually on *Amel*OR109. Six out of the 30 tested odorants were found to activate the receptor (Fig. 1F; Friedman ANOVA, χ^2^ = 142.7, df = 29, *p* < 2.2E-16). These included three compounds belonging to the alarm pheromone. Indeed, IPA was the odorant that triggered the highest response (Fig. 1F; Wilcoxon post hoc test vs mineral oil, *p* = 8.7E-05). (Z)-3-hexenyl acetate (Fig. 1F; *p* = 0.018) and benzyl acetate (Fig. 1F; *p* = 0.02) also activated the receptor. In addition, two compounds from the aggregation pheromone were strongly active: geranic acid (Fig. 1F; *p* = 0.008) and (E,E)-farnesol (Fig. 1F; *p* = 0.007). Finally, linolenic acid, a compound of the queen retinue pheromone, also activated this receptor (Fig. 1F; *p* = 0.008).

### Specificity of *Amel*OR136 and *Amel*OR109 responses

**Fig. 2:**
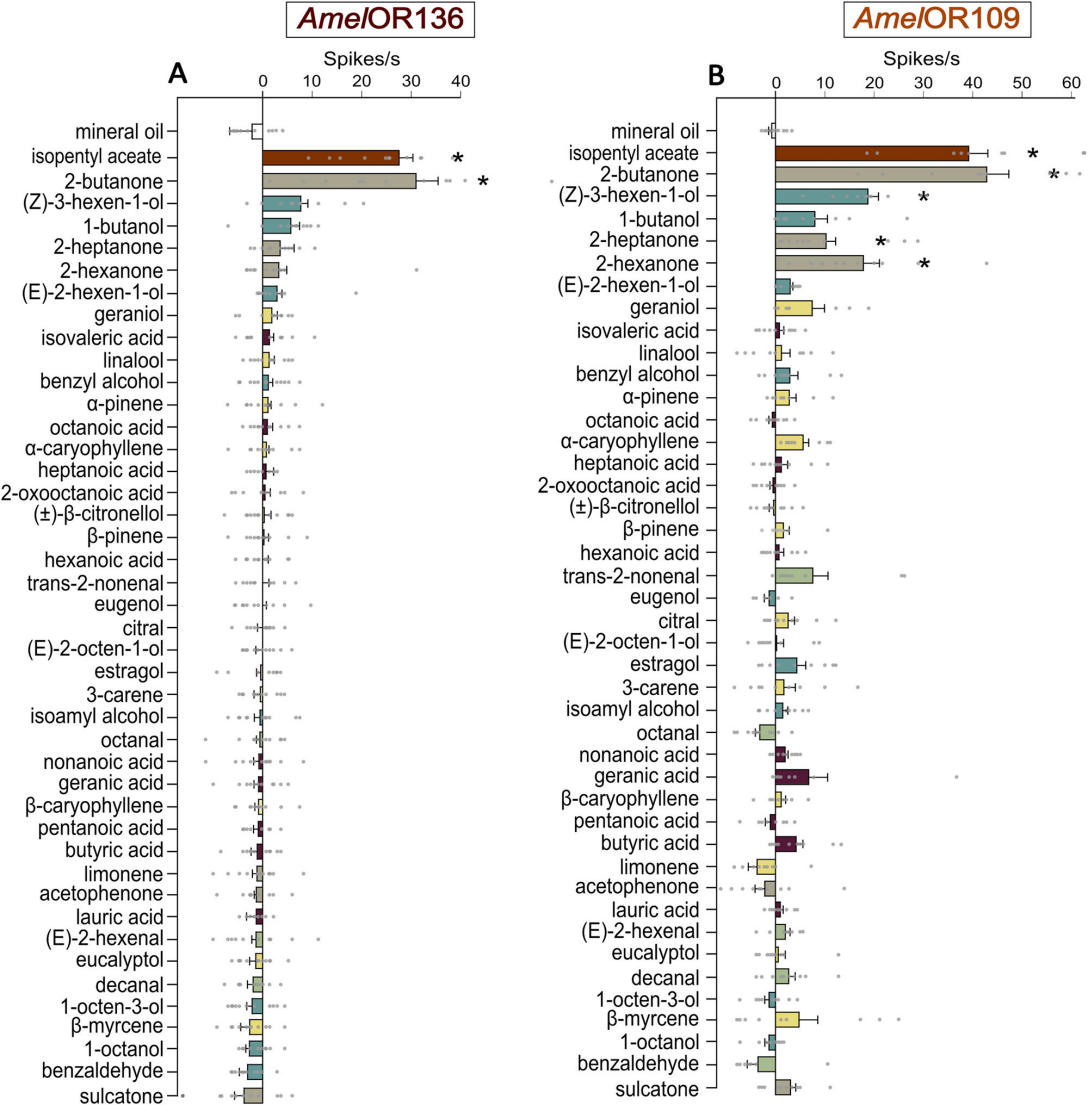
Responses of *Amel*OR136 and *Amel*OR109 to 42 compounds from 6 chemical families measured using single sensillum recording. Mean response of *Amel*OR136 **A** and *Amel*OR109 **B**, in spikes per second, in response to the presentation of 42 compounds presented at 1000 µg. 2-butanone activates *Amel*OR136 (*p < 0.05, *n* = 12). 2-butanone, Z-3-hexen-1-ol, 2-hexanone, and 2-heptanone significantly activate *Amel*OR109 (*p < 0.05, *n* = 10), with some compounds eliciting a minimal response, not reaching the significance threshold but with a different pattern compared to mineral oil.

We then aimed to determine the specificity and sensitivity of these receptors, and used SSR to this end. A panel of 42 novel odorants was tested, representing a wide array of chemical features and aiming to reflect the chemical diversity of odorants that honey bees may encounter in nature. Among the 42 tested odorants, only 2-butanone significantly activated *Amel*OR136 in comparison to the mineral oil control (Fig. 2A; Friedman ANOVA, χ^2^ = 97.5, df = 42, *p* = 2.62 E-06; Wilcoxon post hoc test, *p* = 0.021) with an average activity at around 30 spikes/s. The remaining compounds, including other ketones, did not activate *Amel*OR136 (*p* > 0.1), thus confirming that this receptor is highly specific.

In comparison, *Amel*OR109 was activated by multiple compounds in the panel, including 2-butanone (Fig. 2B; Wilcoxon post hoc test, p = 0.044) and chemically distinct compounds such as (Z)-3-hexen-1-ol (alcohol; Wilcoxon post hoc test, *p* = 0.02), 2-heptanone (*p* = 0.044) and 2-hexanone (*p* = 0.044). A number of other compounds induced modest, not statistically significant response.

**Fig. 3:**
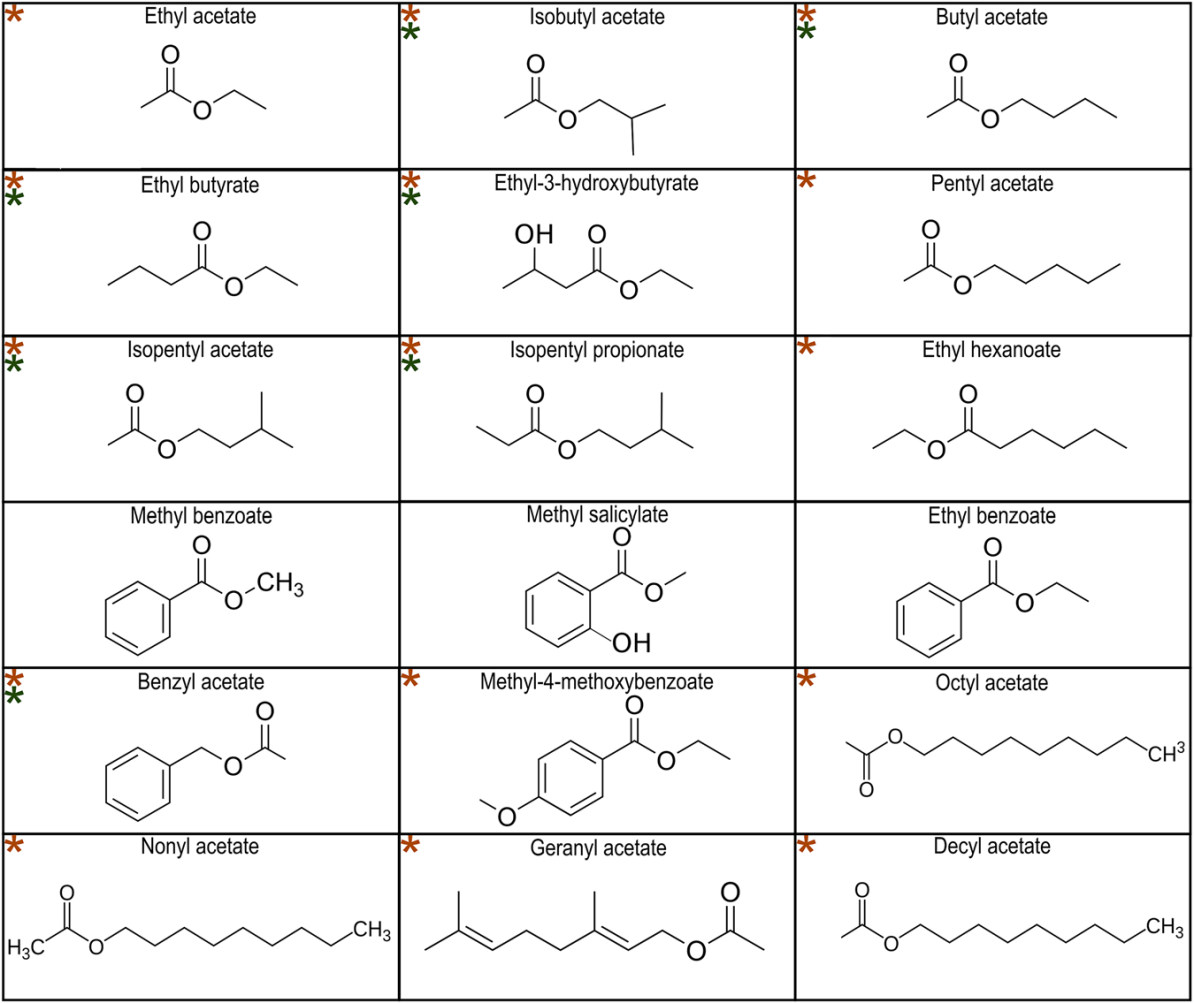
Structure of the ester molecules. Eighteen acetate derivatives were used, ordered from the smallest number of carbons at the top left to the largest number of carbons at the bottom right. A green star indicates molecules activating *Amel*OR136, while orange stars indicate molecules activating *Amel*OR109.

**Fig. 4:**
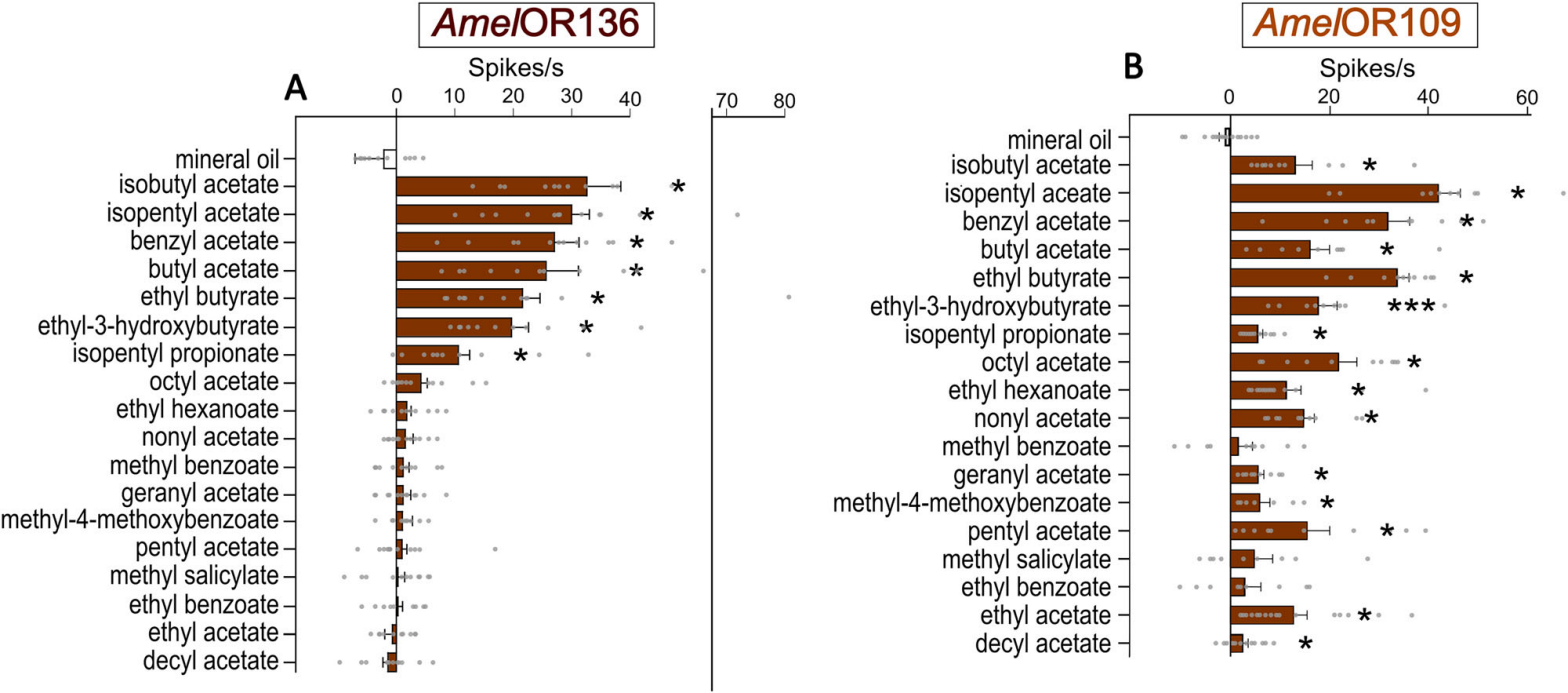
Responses of *Amel*OR136 and *Amel*OR109 to 18 acetate derivatives measured using single sensillum recording. Mean response of *Amel*OR136 **A** and *Amel*OR109 **B**, measured in spikes per second, upon stimulation with one of the 18 acetate derivatives presented at 1000 μg. Isobutyl acetate, isopentyl acetate, benzyl acetate, butyl acetate, ethyl butyrate, ethyl-3-hydroxybutyrate, and isopentyl propionate activate *Amel*OR136 (*p < 0.05, *n* = 12). *Amel*OR109 is activated by 15 ester odorants (*p < 0.05, *n* = 16).

As *Amel*OR136 and *Amel*OR109 were both activated by acetates present in the alarm pheromone (Fig. 1C, F), we proceeded to test the responses of these receptors to a range of different esters varying according to their chain length and structure (Fig. 3). Within this set of 18 esters, 7 compounds activated *Amel*OR136 (Fig. 4A; Friedman ANOVA, χ^2^ = 144.9, df = 19, *p* < 2.2 E-16), including isobutyl acetate, isopentyl acetate, benzyl acetate, butyl acetate, ethyl butyrate, ethyl-3-hydroxybutyrate and isopentyl propionate (in all cases, *p* < 0.003). The response pattern of *Amel*OR109 to the ester panel was distinct from that of *Amel*OR136 and appeared to be more generalist (Fig. 4B; Friedman ANOVA, χ^2^ = 114.1, df = 20, *p* = 3.4 E-15). *Amel*OR109 was significantly activated by 15 out of the 18 esters tested, including the 7 compounds that activated *Amel*OR136 (Wilcoxon post hoc test p < 0.05). Only 3 compounds, methyl benzoate (*p* = 0.64), methyl salicylate (*p* = 0.27) and ethyl benzoate (*p* = 0.4) did not activate this receptor.

Overall, these results suggest that *Amel*OR136 exhibits a more specific response spectrum than *Amel*OR109. To quantify the specificity of *Amel*OR136 and *Amel*OR109 responses, we computed the lifetime sparseness (S), a measure that ranges from 0 (unselective, broadly tuned) to 1 (maximally selective). Using the responses measured to all 61 compounds tested in electrophysiology on both receptors, a lifetime sparseness of 0.67 was obtained for *Amel*OR109, while a higher value of 0.88 was found for *Amel*OR136. This measure corroborates the sparser coding of *Amel*OR136 in comparison to *Amel*OR109.

### Response sensitivity of *AmelOR*136 and AmelOR109

**Fig. 5:**
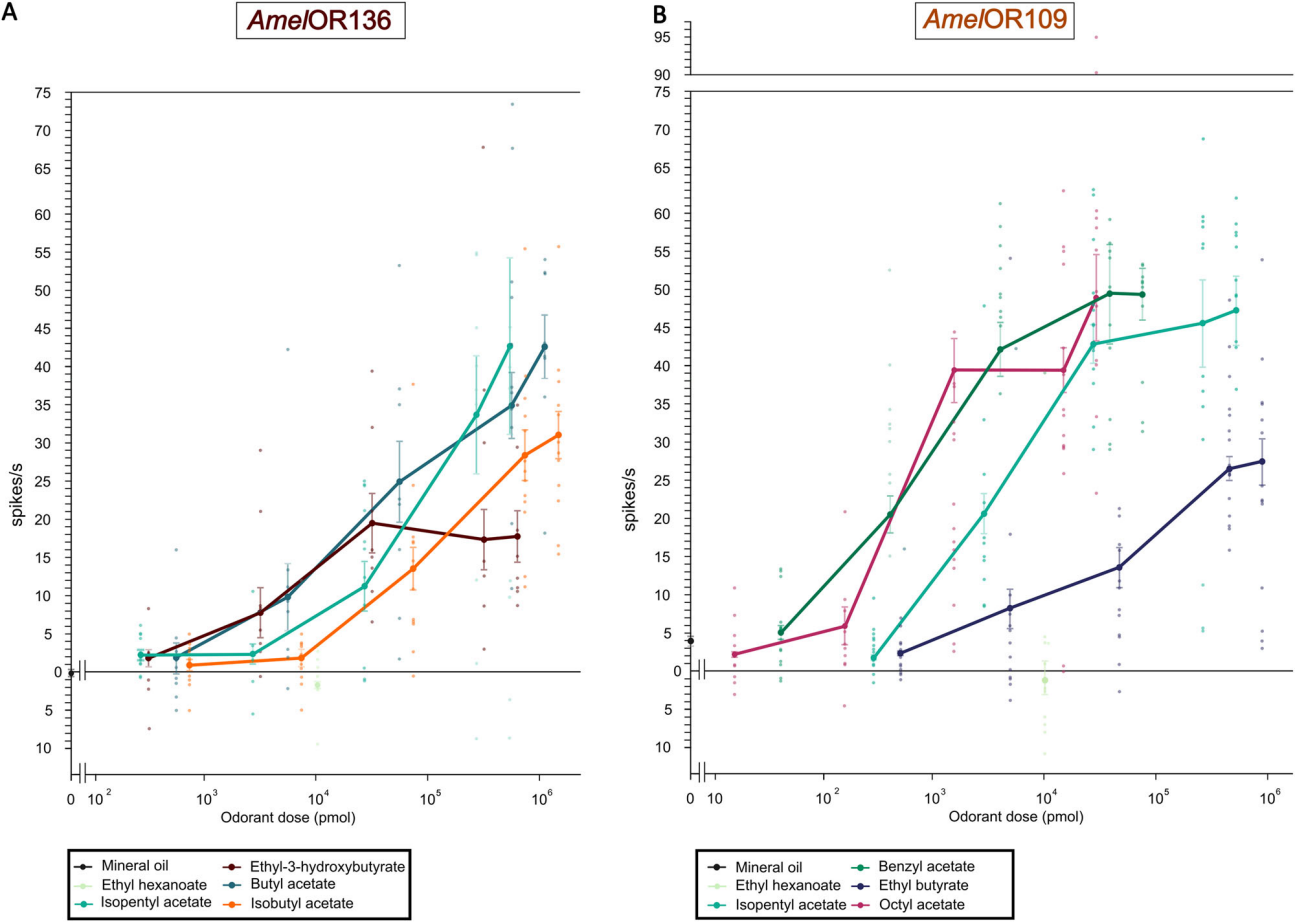
Dose-response curves for *Amel*OR136 and *Amel*OR109 for 4 compounds. Mean response of *Amel*OR136 (**A**, *n* = 13) and *Amel*OR109 (**B**, *n* = 15), in spikes per second. Five different doses of each compound were applied: 1, 10, 100, 1000, and 2000 μg. In order to compare these compounds with different volatilities, the airborne amount of each odorant (in pmol) was calculated using the guidelines in Andersson et al.^39^ (see methods). Responses to mineral oil and ethyl hexanoate (the ligand of *Dme*lOR22a, to ensure that this receptor is not expressed - in light green) are shown as controls.

To approach the sensitivity of the two receptors to their main ligands, we performed dose-response analyses (Fig. 5; from 0 to 2.10^3 ^µg on the filter paper). Four ligands were chosen for each receptor (isobutyl acetate, isopentyl acetate, butyl acetate and ethyl-3-hydroxybutyrate for *Amel*OR136; benzyl acetate, ethyl butyrate, octyl acetate and isopentyl acetate for *Amel*OR109). To allow direct comparisons between odorants, we calculated for each stimulus an estimate of the airborne quantity of odorant in pmol (see Andersson et al.^39^).

We observed a clear increase in the responses of *Amel*OR136 with increasing doses of odorant (GLMM, t = 14.4, *p* < 2 E-16). For each of the four ligands, no significant response was observed at the lowest tested dose (below ${10}^{3}$ pmol). Statistical significance was observed at a dose of 2.7*${10}^{4}$ pmol for isopentyl acetate, 3.2*${10}^{4}$ pmol for ethyl-3-hydroxybutyrate, 5.6*${10}^{4}$ pmol for butyl acetate and 7.4*${10}^{4}$ pmol for isobutyl acetate (Wilcoxon test vs mineral oil, p < 0.01 for each of the four odorants). Notably, responses for ethyl-3-hydroxybutyrate reached a saturation plateau at 3.2*${10}^{4}$ pmol. We conclude that AmelOR136 is most sensitive and responsive to butyl acetate and isopentyl acetate, both compounds of the honey bee alarm pheromone.

For OSNs expressing *Amel*OR109, we also observed a clear increase in responses with increasing doses of odorant (GLMM, t = 13.47, *p* < 2 E-16). Among the four ligands, no significant responses were observed at the lowest tested dose similarly to *Amel*OR136. Statistical significance was observed at a dose of 3.9*${10}^{2}$ pmol for benzyl acetate, 1.5*${10}^{4}$ pmol for octyl acetate, 2.7*${10}^{3}$ pmol for isopentyl acetate and 4.7*${10}^{4}$ pmol for ethyl butyrate (Wilcoxon test vs mineral oil, *p* < 0.02 for each of the four odorants). Responses for isopentyl acetate reached a saturation plateau at 2.7*${10}^{4}$ pmol. We conclude that AmelOR109 is generally more sensitive than AmelOR136, and most sensitive and responsive to acetates in comparison to ethyl butyrate.

## Discussion

In this study, we deorphanized two odorant receptors of the honey bee *Apis mellifera*, *Amel*OR136 and *Amel*OR109, and demonstrated that both responded to social pheromone compounds of the honey bee. These receptors, which belong to the 9-exon clade, were selected based on their expression level, with *Amel*OR136 representing the most highly expressed OR in in-hive bees when compared to foragers^40^, while *Amel*OR109 ranks as one of the most abundantly expressed ORs in the honey bee transcriptome regardless of age or task^34^. We found that these receptors responded to social pheromones but exhibit distinct response spectra: while *Amel*OR136 appeared to respond to a restricted number of compounds, *Amel*OR109 was more broadly tuned, responding to a wider panel of odorants including non-pheromonal compounds.

More specifically, *Amel*OR136 is mainly activated by esters that belong to the alarm pheromone, such as isobutyl acetate, isopentyl acetate (IPA), benzyl acetate and butyl acetate. Alarm pheromones represent the second most prevalent class of chemical signals used by insects, following sex pheromones^41^. The alarm pheromone plays a vital role in alerting and recruiting nestmates, thereby enhancing colony defense. In the honey bee, the alarm pheromone consists of over fifteen chemical compounds^3,11,42^, with IPA representing the most abundant and behaviorally-active compound. IPA has multiple roles: it alerts returning bees to a potential danger, reduces foraging activity^42^, triggers a defensive response and increases aggression against a threat^11,43,44^. The other ester molecules have complementary effects, such as butyl acetate which recruits new defenders, while benzyl acetate is involved in signaling danger to departing and returning foragers^45^. Finally, isopentyl propionate is found on the sting shaft of honey bee workers in several species^46,47^. We observed that *Amel*OR136 exhibits a different sensitivity to the various ester compounds. Our data suggest that the receptor is finely tuned to some of the esters of the alarm pheromone, and remains poorly activated by other odorants. Among these, we observed some responses to compounds produced by queen and worker mandibular glands, 9HDA and 10HDA. Possibly, such sensitivity may play a role in increasing response of the receptor to the alarm pheromone in a colony context. Another compound, the fruit odorant ethyl-3-hydroxybutyrate also activated *Amel*OR136, but responses increased only moderately with increasing doses and remained below those observed for the alarm pheromone esters.

For several decades, most work on the processing of pheromone information in insects has focused on sex pheromones, especially in moths^48–50^. These studies suggested the existence of dedicated, labeled line pathways, in which pheromonal compounds would be detected by highly specific and sensitive ORs at the periphery, and dedicated pathways would convey this information, without much transformation, to specific brain centers, triggering fast and stereotyped behaviors. Given the natural constraints of neural systems (especially the costs associated with the maintenance of a high numbers of neurons), such a system is unable to encode for all potential odorants present in the environment. It has therefore been traditionally considered that other odorants are treated according to a combinatorial across-fiber processing framework^5,51^: each odorant is detected by many, weakly specific ORs, and the identity of a particular odorant can only be extracted by reading out a combination of these channels. In this model, multiple glomeruli are activated in the antennal lobe, and the interpretation of the odorant’s biological significance occurs in higher-order processing centers^5,52–54^. Theoretically, across-fiber processing can code for a much higher number of odorants with the same number of neurons, but it may be less efficient in its ability to detect a particular odorant, especially at low concentration. Overall, *Amel*OR136 is remarkable for its ability to responds to several components of the same pheromone. Such a coding strategy could be highly advantageous for efficiently recognizing the alarm pheromone as a whole and responding quickly, sensitively and efficiently to danger in the complex olfactory environment of the honey bee hive. However, activation of *Amel*OR136 was not exclusive to alarm pheromone compounds, indicating that this receptor is finely tuned but may not be involved in a labeled line coding. Recently, Hart et al.^55^ showed that the coding strategy observed for alarm pheromone in the olfactory system of the clonal raider ant *Ooceraea biroi*, while not entirely adhering to a labeled line model, still employed quite narrowly tuned channels. The features exhibited by *Amel*OR136 in our study may be consistent with these observations.

We found in this study that *Amel*OR109 is activated by ester compounds of the alarm pheromone, but also by several other pheromone blends (aggregation, queen or brood pheromone blends) and numerous individual pheromone compounds with diverse social functions. In contrast to *Amel*OR136, *Amel*OR109 appears to be a generalist receptor (confirmed by the sparseness value, S = 0.67 for *Amel*OR109, against S = 0.88 for *Amel*OR136), and could serve as a secondary contributor in the detection of the alarm pheromone. It is generally assumed that the majority of receptors in honey bees’ repertoires are generalists, involved in combinatorial coding, as most AL glomeruli are known to respond to a relatively wide panel of different odorants^51,56^. As such, *Amel*OR109 responded to several compounds, including (Z)-3-hexen-1-ol, a compound emitted by propolis^57^, or to ethyl hexanoate, a compound associated with varroa infection^58^. Thus, this receptor may play a role in the detection of important pheromones compounds while also contributing to the detection of other odors within or outside the colony.

Both *Amel*OR136 and *Amel*OR109 belong to the 9-exon clade, a subfamily known for its high level of recent gene duplications and sequential evolution compared to other OR subfamilies, initially observed in ants^59^. In honey bees, this subfamily constitutes 24% of the total number of ORs (42 ORs)^40^, whereas it constitutes 30% of the receptors in ants^59^. This 9-exon family is known to play a role in the detection of cuticular hydrocarbons (CHC) in ants^60^, therefore representing the foundation for nestmate recognition and kinship identity in ants, bees and wasps^2^. In this study, we focused on *Amel*OR109 which belongs to the 9-exon beta clade, whereas *Amel*OR136 originates from the 9-exon alpha clade, associated with CHC detection in ants^61^. Previous studies have shown that most 9-exon ORs in *Harpegnathos saltator* exhibit narrow tuning profiles similar to *Amel*OR136, although some are slightly more broadly tuned, in line with the response profile of *Amel*OR109^26,60^.

Interestingly, our findings evidenced that both receptors in honey bees were particularly sensitive to volatile social pheromone compounds, suggesting that members of the 9-exon clade may have evolved diverse functions beyond CHC detection. To further investigate this possibility, we tested whether these receptors could be activated by bee-derived CHC compounds. Although some responses were observed, they remained near the threshold of statistical significance (see Supplementary Fig. 1), suggesting that *Amel*OR136 and *Amel*OR109 are not primarily involved in the detection of CHCs in *Apis mellifera*. Note that nevertheless *Amel*OR136 exhibited a small affinity for the unsaturated hydrocarbons nonadecene and heneicosane (see Supplementary Fig. 1). Such functional versatility of ORs could allow the honey bee to integrate volatile and contact-mediated signals in a context-dependent manner, as recently suggested by Mondet et al.^62^. The absence of response of *AmelOR*109 to the tested CHCs may reflect functional specializations within the 9-exon clade, whereby certain receptors respond preferentially to volatile pheromones, while others — yet to be identified — may preferentially detect CHCs. Alternatively, the apparent lack of response to CHCs in our preliminary data may also result from technical limitations of the expression system: CHCs are heavy, low-volatility molecules with high molecular weight, which limits their effective diffusion and availability in our stimulation setup. It is therefore possible that the concentration reaching the receptor-expressing neurons remained below the detection threshold^39^.

In Hymenoptera, there is no compelling evidence for a strict segregation of OR subfamilies in the detection of CHCs^26,63^. This suggests that other ORs, in addition to the 9-exon ORs, may be sensitive to CHCs^2^. Consequently, it can be postulated that 9-exon ORs in honey bees may be involved in a wider range of functions than CHC detection. A more comprehensive survey of ORs from this clade is necessary to determine whether some are indeed specialized in CHC detection, while others may be involved in the detection of bees’ other social pheromones.

Crucially, the Drosophila-based system provides fine ligand-profiling, but in vivo validation of the response of OSNs expressing our receptors via single-sensillum recordings (SSR) on *Apis mellifera* antennae^64,65^ would be important. However, honey bee placode sensilla typically contain 5-35 OSNs, which makes the identification of the responses of individual neurons electrophysiologically very difficult^66^. It would be also interesting to use in situ hybridization or immunohistochemistry to determine if *Amel*OR136 and *Amel*OR109 co-localize within the same sensillum or are segregated in different sensilla. Presence in the same sensillum could leverage within-sensilla processing for enhanced detection of alarm pheromone, but this will need to be determined.

Further studies at the level of the antennal lobe could also contribute to a more comprehensive understanding of how information about odorants detected by these receptors is encoded in the brain. Although exceptions have been observed (e.g. Herre et al.^67^), OSNs expressing the same OR generally converge in a common glomerulus. Using in vivo optical imaging, one may be able to identify the glomeruli receiving OSNs that express *Amel*OR136 and *Amel*OR109 by presenting the same panels of odorants tested in this study. We can anticipate that the response profiles of these glomeruli will closely match those of the two receptors, as measured here in a heterologous expression system. Such experiments would also be important for addressing the question of whether a labeled line for alarm pheromone exists in the honey bee brain. Theoretically, it is expected that labeled lines are relatively isolated from the rest of the olfactory system, thus ensuring that the response to the pheromone remains sensitive and fast, protected from interference by background odors present in the environment^5,68^. As done in previous studies^69,70^, it should be possible to evaluate to which extent processing by local networks within the antennal lobe affects the transmission of information from *Amel*OR136 or *Amel*OR109 OSNs. Furthermore, studies could be conducted to investigate the impact of these receptors on behavior. Recent developments in genetic editing have introduced the possibility to develop honey bee neurogenetic tools, with the GCaMP-expressing bees^71^ serving as a pioneering example. CRISPR/Cas9 has revolutionized functional genetics in insects—and has been successfully applied to silence ORCO or individual ORs, for instance in *Spodoptera littoralis*, *Bombyx mori*, *Manduca sexta, Locusta migratoria*^72–75^, and, critically, to ORCO in *Apis mellifera*^76^. CRISPR/Cas9 genome editing in honey bees faces challenges (low transformation rates, unknown neuronal promoters, queen survival), but recent advances in embryo microinjection and germline delivery have markedly improved editing efficiency^71^. The generation of transgenic bees that do not express *Amel*OR136 seems achievable in the near future and would be a crucial step towards elucidating the possible role of this receptor in honey bees’ alarm and defense behaviors. It is hypothesized that the absence of this receptor will have a significant impact on bees’ behavioral response to the alarm pheromone, as well as on their efficiency in aggression tests^77^.

In conclusion, we have functionally characterized two new ORs in the honey bee *Apis mellifera*. We showed that *Amel*OR136 and *Amel*OR109 respond to social pheromonal compounds. Whereas *Amel*OR136 presented a sparse response spectrum, mainly responding to alarm pheromone esters, *Amel*OR109 appeared to be more generalist, responding to a wide range of odorants. This work represents a significant step forward in understanding the neural encoding of social pheromone information in the brains of social insects.

## Methods

### OR selection

In order to identify receptors potentially sensitive to social pheromones, we selected ORs based on their expression level in bees’ antennae and their phylogenetic position, using published data^33,34,78,79^. A list of 8 ORs (see list in Supplementary Data 1) overexpressed in female workers in comparison to males^34^ was selected, as workers play a more prominent role in behaviors involving the detection of social pheromones. We also considered ORs that were overexpressed in in-hive bees compared to foragers^40^, since the former are expected to be more strongly exposed to social pheromones than the latter. Experiments were performed at EGCE Lab in Gif-sur-Yvette (France) between 2021 and 2025.

### Heterologous expression of *Amel*ORs in Drosophila

The peripheral olfactory system of bees is not well suited to the specific study of individual OSNs, as a single olfactory sensillum (placode) can house up to 35 different OSNs^64,80^. To overcome this limitation, we used heterologous expression of ORs in the *Drosophila melanogaster* empty neuron system^81^, commonly used for deorphanizing ORs of Lepidoptera^24^ but also some Hymenoptera^26,60,82^. All honey bee ORs (*Amel*ORX, sequences in Supplementary Data 1) were expressed in ab3A OSNs of *D. melanogaster* (housed in basiconic sensilla) lacking the endogenous receptors *Dmel*OR22a and *Dmel*OR22b. To do so, the full-length open reading frames of *Amel*ORX were synthesized in vitro using codon optimization for expression in *Drosophila* and were inserted into the pUAST.attB vector (Synbio Technologies, Monmouth Junction, NJ, USA). Balanced UAS-*Amel*ORX flies were generated by Bestgene Inc. (Chino Hills, CA, USA), by injecting the pUAST.attB-*Amel*ORX plasmid (Endofree preparation) into fly embryos with the genotype y[1] M{vas-int.Dm}ZH-2A w[*]; M{3xP3-RFP.attP}ZH-86Fb, leading to a non-random insertion of the UAS-*Amel*ORX construct into the locus 86F8 of the third chromosome. Subsequently, each UAS-*Amel*ORX line was crossed with the *Dmel*OR22ab^GAL4^ line^83^ to obtain homozygous flies with the genotype *w; Dmel*OR22ab^*GAL4*^*; UAS-Amel*ORX. Flies were reared on standard cornmeal-yeast-agar medium and kept in the dark in a climate-controlled environment (25 °C, 27% humidity). Effective presence of the *UAS-Amel*ORX transgenes and absence of the *Dmel*OR22ab gene were verified using PCR on genomic DNA extracted from 10 flies (Primers in Supplementary Table 1). Prior to calcium imaging experiments, flies expressing *Amel*ORs were crossed with flies expressing the UAS-GCaMP6s^84^ calcium indicator to generate heterozygous flies with the genotype *w; Dmel*OR22ab^GAL4^*; UAS-Amel*ORX*/UAS-GCaMP6s*.

### Fly containment

To access the region of the antenna where ab3 basiconic sensilla are located, 3- to 7-day old flies were gently secured at the narrow end of a 200 μL plastic pipette tip, allowing only the antennae and half of the head to protrude. The tip was then securely fixed on a microscope glass slide using dental wax, exposing the ventral side of the fly upwards. One antenna was gently pressed onto a piece of microscope slide using a glass capillary positioned between the pedicel and the funiculus. The glass capillary itself was firmly secured using low-temperature melting wax, ensuring that the antenna remained immobile throughout the experiment^85^. A total of 75 flies expressing *Amel*0R136 and 61 flies expressing *Amel*0R109 were used in this study, the minimum number of individuals for assuring statistical robustness of the data.

### Single sensillum recordings (SSR)

Mounted flies were placed under a microscope (BX51Wl, Olympus, Tokyo, Japan) equipped with an Olympus SLMPlan 100X/0.6 objective. Action potentials were recorded from the ab3A OSNs using tungsten electrodes (Phymep, Paris, catalog number: 716000), which were sharpened using electrolysis in a 10% potassium hydroxide solution. The reference electrode was inserted into the fly’s eye using a mechanical micromanipulator (Leitz, Wetzlar, Germany), and the recording tungsten electrode was inserted at the base of the sensillum using a motorized micromanipulator (PatchStar, Scientifica Ltd, UK). The electrical signal was amplified 10 times, then filtered (1 Hz -3 kHz) and further amplified (x50) with an AC-DC amplifier (EX1 Differential Amplifier, Dagan Corporation USA). The data were acquired on a computer by sampling at 10 kHz for 15 s with a precision of 16 bits. A data acquisition card (DT 9813, Data Translation, USA) was used, driven by the dbWave software^86^. The timing of the stimulation was recorded on a second channel using a TTL signal generated by the olfactory stimulation device.

### Calcium imaging

Optical imaging recordings on fly antennae were performed using an Olympus BX51WI microscope with a Zeiss LD A-Plan 10x/0.25 Ph1 objective. The observed area was captured by an Evolve® 512 camera (Photometrics, 16 bits, binning 1:1). The recordings were made using routines implemented in Visiview® Software (Visitron Systems GmbH, Puchheim, Germany). A monochromator produced excitation light at 488 nm (Polychrome 5000, Till Photonics, Martinsried, Germany). Emission was collected using a 525BP50 filter. Each recording consisted of 100 frames (80 ms exposure time) of 512 ×512 pixels at a frequency of 5 Hz, which were saved as an STK file. Each recording thus lasted 20 s. A 90 s interval was applied between recordings. The first recording was performed without any stimulus, to measure fluorescence decay over the course of the recording (photobleaching). The collected data was used for photobleaching correction^38^.

### Olfactory stimulation

Throughout the experiments, flies were exposed to a constant airflow of 3 L/min. During stimulation, a fraction of the air flow (500 mL/min) was redirected through the stimulation cartridge. Each stimulation cartridge consisted of a Pasteur pipette containing a 1 cm^2^ piece of filter paper soaked with 10 μL of the odorant solution. Each odorant was presented only once per individual.

For electrophysiological recordings, the stimulation was applied for 1 s, starting 5 s after the beginning of a recording. For calcium imaging recordings, the stimulation also lasted for 1 s, but started after 3 s (15^th^ to 20^th^ frame).

At the beginning of each experiment, control stimuli were presented: mineral oil, used as a solvent control, and ethyl hexanoate, the main ligand of *Dmel*OR22a, the endogenous receptor expressed in ab3A neurons of the fruit fly. This last recording aimed to ensure the absence of the *Drosophila* receptor in the recorded individual. In electrophysiological experiments, 2-heptanone, the main ligand of *Dmel*OR85b (expressed in the second OSN present in ab3a sensilla) was also presented to verify that the recorded sensillum was indeed an ab3. Odorants were always presented in a randomized order.

### Odorant stimuli

In order to identify ORs tuned to social pheromones, a series of six blends was presented (OSN activity recorded using calcium imaging), each containing the main compounds of honey bees’ major pheromones (Table 1, pure odorants, equal volume of each odorant in the blend).

**Table 1:** Compounds of the pheromonal blends

Blend	Compounds	References
*“Alarm pheromone”*	octyl acetate (Z)-3-hexenyl acetate IPA (isopentyl acetate) 2-heptanone 2-nonanol (Z)-11-eicosen-1-ol	^10,42,43,114^
*“Queen Mandibular Pheromone* (QMP)”	HVA (9-hydroxy-2-decenoic acid) HOB (4-hydroxy-3-methoxyphenylethanol) 9-ODA (9-oxo-2-decenoic acid) 9-HDA (9-hydroxy-2-decanoic acid) 10-HDA (10-hydroxy-2-decenoic acid)	^115–117^
*“Queen Retinue Pheromone* (QRP)”	linolenic acid hexadecan-1-ol coniferyl alcohol methyl salicylate	^118^
*“Aggregation pheromone”*	citral nerol geranic acid (E,E)-farnesol	^87,119,120^
*“Waggle dance pheromone”*	n-pentacosane n-tricosane (Z)-9-tricosene	^13^
*“Brood pheromone”*	methyl linoleate ethyl palmitate methyl linolenate ethyl oleate stearic acid β-ocimene	^15,121^

In addition to the pheromonal blends, flies were exposed to a panel of 42 odorants (OSN activity recorded in SSR), comprising both pheromonal compounds and floral scents relevant to honey bee’s lifestyle. These odorants were identified in the literature as being present in honey bees’ natural environment and/or previously shown to be detected by bees^9–11,16,25,42,44,46,47,57,69,87–108^. We ensured chemical diversity by including compounds from different functional groups (e.g., alcohols, aldehydes, acids, esters, and terpenes) and with varied carbon chain lengths. Furthermore, we used molecular descriptors from the PubChem database to optimize distribution across the chemical space. This allowed to assess the specificity of each *Amel*ORX.

Subsequently, a second, more specific panel was presented to the flies, comprising 18 odorants belonging to the ester class, as *Amel*OR109 and *Amel*OR136 were found to be highly responsive to isopentyl acetate. This panel included short-chain esters (e.g., ethyl acetate [4 C], isobutyl acetate [6 C], butyl acetate [6 C]), medium-chain esters (e.g., isopentyl acetate [7 C], benzyl acetate [9 C], octyl acetate [10 C]), and long-chain esters (e.g., nonyl acetate [11 C], geranyl acetate [12 C], decyl acetate [12 C], and farnesyl acetate [17 C]). New stimulation cartridges were prepared daily. All chemicals were obtained from Thermofisher Scientific (Waltham, United States) except for 9-ODA bought from Apollo scientific (purity 95%, Bredbury, UK). All odorants were presented at a concentration of 100 μg/μL (diluted in mineral oil). A minimum of 10 flies were tested for each odorant.

For some of the receptors’ ligands, dose-response experiments were conducted using concentrations ranging from 0.1 μg/μL to 1000 μg/μL. We focused these dose–response curves on ethyl-3-hydroxybutyrate, butyl acetate, isobutyl acetate and isopentyl acetate for *Amel*OR136, and benzyl acetate, ethyl butyrate, octyl acetate and isopentyl acetate for *Amel*OR109. These compounds were among those that elicited the strongest and most reproducible responses in OSNs expressing these receptors. To compensate for differences in volatility in dose-response experiments, we calculated for each stimulus concentration, an estimate of the airborne quantity of molecules flowing out of the stimulus cartridge, following the guidelines formulated by Andersson et al.^39^. For a 100-µg load of odorant on the filter paper, the depletion rate Y was calculated, as Y = -0.010 BP – 0.178 L + 0.519, with *BP* the odorant’s boiling point, and *L* its lipophilicity. Then a log-log regression of the data presented in Fig. 4A of the study by Andersson et al.^39^ was used, as it provides a better fit to the data (R^2^ = 0.91) and to the assumptions of linear regression than the log-linear approach presented in the article (Fredrik Schlyter, pers. comm.). The estimated dose in the airflow could thus be calculated as follows:

$${{\rm{Log\; Dose}}}\left({{\rm{pmol}}}\right)=\frac{Y+4.7606}{0.7797}$$

### Data Analysis

For calcium imaging experiments, we calculated the variation of fluorescence intensity during each recording using the following formula: $\Delta {{\rm{F}}}=\frac{{{\rm{F}}}({{\rm{t}}})-{{\rm{F}}}(0)}{{{\rm{F}}}(0)}$ where F(0) corresponds to the average of 5 frames just before odor stimulation (frames 11 to 15). Photobleaching was corrected by subtracting from each recording the fluorescence decay measured in the first recording without any stimulus (see above). The quantification of response intensity was obtained by averaging frames following odor presentation (frames 17 to 31) and subtracting the average before odor presentation (frames 11 to 15).

For SSR, peri-stimulus time histograms were generated by dividing the 15 s recording into 100 ms bins. The average number of spikes per bin for all odorants tested was calculated. We observed that, on average, the odor responses occurred between 6.3 s and 8.1 s, with a peak response at 7 seconds. Thus, the responses of ab3A neurons were analyzed by subtracting the spontaneous firing rate (measured during 1.8 seconds before the onset of stimulation, in spikes per second) from the firing rate induced by odorant stimulation, calculated by averaging the number of spikes during a 1.8-second window (from 6.3 s to 8.1 s).

### Statistics and Reproducibility

Responses to odorant stimuli were compared statistically to the control without odorant (air for pheromonal blends or mineral oil for the other experiments). The normality of the data distribution was evaluated using a density diagram and a Quantile-Quantile (QQ) plot. As the data did not follow a normal distribution, we compared responses to the different odorants in a panel using a Friedman test, followed by Wilcoxon post-hoc tests to compare response intensities between odorants and their respective controls. Due to multiple comparisons, the significance threshold was corrected using the Benjamini-Hochberg false discovery rate correction method. The odorants were considered active if the response was statistically higher than the response elicited by the control alone. All statistical tests were performed using R version 4.0.3.

For the dose-response experiments, a Generalized Linear Mixed-Effects Models (*glmer* function^109^) with two main predictors was employed: *Odorant* (representing the different odorants tested) and *Dose*. The model also considers individual-specific variations by including a random intercept for each individual. The response variable followed a gamma distribution, and we used a logarithm link function. The interaction term between *Odorant* and *Dose* was added in the model. Responses below 0 do not allow the use of Gamma family, so a constant was added to all data to ensure all values were positive.

The specificity of a receptor’s response spectrum was calculated using the *lifetime sparseness* formula^110–112^:

$$s=\left(\frac{1}{1-\frac{1}{n}}\right)\times \left(1-\left({\left(\sum \limits_{i=1,n}{r}_{i}/n\right)}^{2}/\sum\limits_{i=1,n}\left({{r}_{i}}^{2}/n\right)\right)\right)$$

With ${r}_{i}$ the amplitude of the response to stimulus i in the panel of n stimuli. The lifetime sparseness (s) ranges from 0 (unselective, broadly tuned) to 1 (maximally selective).

### Reporting summary

Further information on research design is available in the Nature Portfolio Reporting Summary linked to this article.

## Supplementary information

Supplementary Material

Description of Additional Supplementary Files

Supplementary Data 1

Reporting Summary

## Data Availability

The experimental data from calcium imaging and single-sensillum recordings were deposited into Dryad^113^ and are available at the following URL: 10.5061/dryad.rv15dv4k2. Raw data are accessible in this repository, and all other data are available from the corresponding author upon reasonable request.

## References

[1] Aron, S. & Passera, L. *Les sociétés animales: Évolution de la coopération et organisation sociale*. (De Boeck Supérieur, 2009).

[2] Couto, A. et al. Evolution of the neuronal substrate for kin recognition in social Hymenoptera. *Biol. Rev.* **98**, 2226–2242 (2023). 37528574 10.1111/brv.13003

[3] Slessor, K., Winston, M. & Le Conte, Y. Pheromone Communication in the Honeybee (Apis mellifera L.). *J. Chem. Ecol.* **31**, 2731–2745 (2005). 16273438 10.1007/s10886-005-7623-9

[4] Leonhardt, S. D., Menzel, F., Nehring, V. & Schmitt, T. Ecology and Evolution of Communication in Social Insects. *Cell* **164**, 1277–1287 (2016). 26967293 10.1016/j.cell.2016.01.035

[5] Haverkamp, A., Hansson, B. S. & Knaden, M. Combinatorial Codes and Labeled Lines: How Insects Use Olfactory Cues to Find and Judge Food, Mates, and Oviposition Sites in Complex Environments. *Front. Physiol*. **9**, (2018). 10.3389/fphys.2018.00049 PMC5799900 29449815

[6] Mumoki, F. N. & Crewe, R. M. Pheromone communication in honey bees (Apis mellifera). In *Insect Pheromone Biochemistry and Molecular Biology* 183–204 (Elsevier, 2021).

[7] Trhlin, M. & Rajchard, J. Chemical communication in the honeybee (Apis mellifera L.). *a review. Veterinární Medicína* **56**, 265–273 (2011).

[8] Mariette, J., Carcaud, J. & Sandoz, J.-C. The neuroethology of olfactory sex communication in the honeybee Apis mellifera L. *Cell Tissue Res*. **383**, 177–194 (2021). 33447877 10.1007/s00441-020-03401-8

[9] Collins, A. & Blum, M. Bioassay of Compounds Derived from the Honeybee Sting. *J. Chem. Ecol.* **8**, 463–470 (1982). 24414957 10.1007/BF00987794

[10] Pickett, J. A., Williams, I. H. & Martin, A. P. Z)-11-eicosen-1-ol, an important new pheromonal component from the sting of the honey bee,Apis mellifera L. (Hymenoptera, Apidae.). *J. Chem. Ecol.* **8**, 163–175 (1982). 24414592 10.1007/BF00984013

[11] Boch, R., Shearer, D. A. & Stone, B. C. Identification of isoamyl acetate as an active component in the sting pheromone of the honey bee. *Nature* **195**, 1018–1020 (1962). 13870346 10.1038/1951018b0

[12] Millor, J., Pham-Delegue, M., Deneubourg, J. L. & Camazine, S. Self-organized defensive behavior in honeybees. *Proc. Natl. Acad. Sci. USA* **96**, 12611–12615 (1999). 10535970 10.1073/pnas.96.22.12611 PMC23012

[13] Thom, C., Gilley, D. C., Hooper, J. & Esch, H. E. The Scent of the Waggle Dance. *PLoS Biol*. **5**, e228 (2007). 17713987 10.1371/journal.pbio.0050228 PMC1994260

[14] Le Conte, Y., Mohammedi, A. & Robinson, G. E. Primer effects of a brood pheromone on honeybee behavioural development. *Proc. Biol. Sci.* **268**, 163–168 (2001). 11209886 10.1098/rspb.2000.1345 PMC1088586

[15] Maisonnasse, A., Lenoir, J.-C., Beslay, D., Crauser, D. & Conte, Y. L. E. - β-Ocimene, a Volatile Brood Pheromone Involved in Social Regulation in the Honey Bee Colony (Apis mellifera). *PLoS ONE* **5**, e13531 (2010). 21042405 10.1371/journal.pone.0013531 PMC2958837

[16] Noël, A. et al. Detailed chemical analysis of honey bee (Apis mellifera) worker brood volatile profile from egg to emergence. *PLoS ONE* **18**, e0282120 (2023). 36809298 10.1371/journal.pone.0282120 PMC9943000

[17] Hansson, B. S. & Stensmyr, M. C. Evolution of insect olfaction. *Neuron* **72**, 698–711 (2011). 22153368 10.1016/j.neuron.2011.11.003

[18] Hallberg, E. & Hansson, B. S. Arthropod sensilla: morphology and phylogenetic considerations. *Microsc. Res. Tech.* **47**, 428–439 (1999). 10607382 10.1002/(SICI)1097-0029(19991215)47:6<428::AID-JEMT6>3.0.CO;2-P

[19] Vogt, R. G. Molecular basis of pheromone detection in insects. *Compr. Insect Physiol. Pharmacol. Mol. Biol.* **3**, 753–804 (2005).

[20] Clyne, P. J. et al. A novel family of divergent seven-transmembrane proteins: candidate odorant receptors in Drosophila. *Neuron* **22**, 327–338 (1999). 10069338 10.1016/s0896-6273(00)81093-4

[21] Vosshall, L. B., Wong, A. M. & Axel, R. An olfactory sensory map in the fly brain. *Cell* **102**, 147–159 (2000). 10943836 10.1016/s0092-8674(00)00021-0

[22] Benton, R., Sachse, S., Michnick, S. W. & Vosshall, L. B. Atypical Membrane Topology and Heteromeric Function of Drosophila Odorant Receptors In Vivo. *PLOS Biol* **4**, e20 (2006). 16402857 10.1371/journal.pbio.0040020 PMC1334387

[23] Carey, A. F., Wang, G., Su, C.-Y., Zwiebel, L. J. & Carlson, J. R. Odorant reception in the malaria mosquito Anopheles gambiae. *Nature* **464**, 66–71 (2010). 20130575 10.1038/nature08834 PMC2833235

[24] de Fouchier, A. et al. Functional evolution of Lepidoptera olfactory receptors revealed by deorphanization of a moth repertoire. *Nat. Commun.* **8**, 15709 (2017). 28580965 10.1038/ncomms15709 PMC5465368

[25] Hallem, E. A. & Carlson, J. R. Coding of Odors by a Receptor Repertoire. *Cell* **125**, 143–160 (2006). 16615896 10.1016/j.cell.2006.01.050

[26] Slone, J. D. et al. Functional characterization of odorant receptors in the ponerine ant, Harpegnathos saltator. *Proc. Natl. Acad. Sci. USA* **114**, 8586–8591 (2017). 28696298 10.1073/pnas.1704647114 PMC5559025

[27] Wang, G., Carey, A. F., Carlson, J. R. & Zwiebel, L. J. Molecular basis of odor coding in the malaria vector mosquito Anopheles gambiae. *Proc. Natl. Acad. Sci. USA* **107**, 4418–4423 (2010). 20160092 10.1073/pnas.0913392107 PMC2840125

[28] Bicker, G., Kreissl, S. & Hofbauer, A. Monoclonal antibody labels olfactory and visual pathways in Drosophila and Apis brains. *J. Comp. Neurol.* **335**, 413–424 (1993). 8227528 10.1002/cne.903350310

[29] Gascuel, J. & Masson, C. Developmental study of afferented and deafferented bee antennal lobes. *J. Neurobiol.* **22**, 795–810 (1991). 1779223 10.1002/neu.480220802

[30] Benton, R. *Drosophila* olfaction: past, present and future. *Proc. R. Soc. B Biol. Sci.* **289**, 2022–2054 (2022). 10.1098/rspb.2022.2054 PMC9748782 36515118

[31] Paoli, M. & Galizia, G. C. Olfactory coding in honeybees. *Cell Tissue Res*. **383**, 35–58 (2021). 33443623 10.1007/s00441-020-03385-5 PMC7873095

[32] Sandoz, J. C. Behavioral and neurophysiological study of olfactory perception and learning in honeybees. *Front. Syst. Neurosci.* **5**, 98 (2011). 22163215 10.3389/fnsys.2011.00098 PMC3233682

[33] Robertson, H. M. & Wanner, K. W. The chemoreceptor superfamily in the honey bee, Apis mellifera: expansion of the odorant, but not gustatory, receptor family. *Genome Res.* **16**, 1395–1403 (2006). 17065611 10.1101/gr.5057506 PMC1626641

[34] Jain, R. & Brockmann, A. Sex-specific molecular specialization and activity rhythm-dependent gene expression in honey bee antennae. *J. Exp. Biol*. **223**, (2020). 10.1242/jeb.217406 32393545

[35] Claudianos, C. et al. Odor memories regulate olfactory receptor expression in the sensory periphery. *Eur. J. Neurosci.* **39**, 1642–1654 (2014). 24628891 10.1111/ejn.12539

[36] Mariette, J. et al. Evolution of queen pheromone receptor tuning in four honeybee species (Hymenoptera, Apidae, Apis). *iScience* **27**, 111243 (2024). 39610706 10.1016/j.isci.2024.111243 PMC11602622

[37] Wanner, K. W. et al. A honey bee odorant receptor for the queen substance 9-oxo-2-decenoic acid. *Proc. Natl. Acad. Sci. USA* **104**, 14383–14388 (2007). 17761794 10.1073/pnas.0705459104 PMC1964862

[38] Mariette, J. et al. Transcuticular calcium imaging as a tool for the functional study of insect odorant receptors. *Front. Mol. Neurosci*. **16**, (2023). 10.3389/fnmol.2023.1182361 PMC10461100 37645702

[39] Andersson, M. N., Schlyter, F., Hill, S. R. & Dekker, T. What Reaches the Antenna? How to Calibrate Odor Flux and Ligand–Receptor Affinities. *Chem. Senses* **37**, 403–420 (2012). 22362868 10.1093/chemse/bjs009

[40] Zhou, X. et al. Chemoreceptor evolution in hymenoptera and its implications for the evolution of eusociality. *Genome Biol. Evol.* **7**, 2407–2416 (2015). 26272716 10.1093/gbe/evv149 PMC4558866

[41] Barbier. *Les Phéromones: Aspects biochimiques et biologiques*. (Dunod, 1982).

[42] Wager, B. R. & Breed, M. D. Does honey bee sting alarm pheromone give orientation information to defensive bees? *Ann. Entomol. Soc. Am.* **93**, 1329–1332 (2000).

[43] Collins, A. & Kubasek, K. Field Test of Honey Bee (Hymenoptera: Apidae) Colony Defensive Behavior. *Ann. Entomol. Soc. Am.* **75**, 383–387 (1982).

[44] Blum, M. S., Fales, H. M., Tucker, K. W. & Collins, A. M. Chemistry of the sting apparatus of the worker honey bee. *J Apic. Res*. **17**, 218–221 (1978).

[45] Wang, Z. & Tan, K. Honey Bee Alarm Pheromone Mediates Communication in Plant–Pollinator–Predator Interactions. *Insects* **10**, 366 (2019). 31640201 10.3390/insects10100366 PMC6835895

[46] Li, J., Wang, Z., Tan, K., Qu, Y. & Nieh, J. C. Effects of natural and synthetic alarm pheromone and individual pheromone components on foraging behavior of the giant Asian honey bee, Apis dorsata. *J. Exp. Biol.* **217**, 3512–3518 (2014). 25104758 10.1242/jeb.110171

[47] Blum, M. S., Fales, H. M., Morse, R. A. & Underwood, B. A. Chemical Characters of Two Related Species of Giant Honeybees (Apis dorsata and A. laboriosa): Possible Ecological Significance. *J. Chem. Ecol.* **26**, 801–807 (2000).

[48] Xu, M. et al. Olfactory perception and behavioral effects of sex pheromone gland components in Helicoverpa armigera and Helicoverpa assulta. *Sci. Rep.* **6**, 22998 (2016). 26975244 10.1038/srep22998 PMC4792173

[49] Kurtovic, A., Widmer, A. & Dickson, B. J. A single class of olfactory neurons mediates behavioural responses to a Drosophila sex pheromone. *Nature* **446**, 542–546 (2007). 17392786 10.1038/nature05672

[50] Lee, S.-G., Carlsson, M. A., Hansson, B. S., Todd, J. L. & Baker, T. C. Antennal lobe projection destinations of Helicoverpa zea male olfactory receptor neurons responsive to heliothine sex pheromone components. *J. Comp. Physiol. A* **192**, 351–363 (2006). 10.1007/s00359-005-0071-8 16308703

[51] Carcaud, J., Giurfa, M. & Sandoz, J.-C. Differential Combinatorial Coding of Pheromones in Two Olfactory Subsystems of the Honey Bee Brain. *J. Neurosci.* **35**, 4157–4167 (2015). 25762663 10.1523/JNEUROSCI.0734-14.2015 PMC6605296

[52] Brandstaetter, A. S. & Kleineidam, C. J. Distributed representation of social odors indicates parallel processing in the antennal lobe of ants. *J. Neurophysiol.* **106**, 2437–2449 (2011). 21849606 10.1152/jn.01106.2010

[53] Galizia, C. G. & Rössler, W. Parallel olfactory systems in insects: anatomy and function. *Annu. Rev. Entomol.* **55**, 399–420 (2010). 19737085 10.1146/annurev-ento-112408-085442

[54] Sandoz, J.-C., Deisig, N., De Brito Sanchez, M. G. & Giurfa, M. Understanding the logics of pheromone processing in the honeybee brain: from labeled-lines to across-fiber patterns. *Front. Behav. Neurosci*. **1**, (2007). 10.3389/neuro.08.005.2007 PMC2525855 18958187

[55] Hart, T. et al. Sparse and stereotyped encoding implicates a core glomerulus for ant alarm behavior. *Cell* **186**, 3079–3094.e17 (2023). 37321218 10.1016/j.cell.2023.05.025 PMC10334690

[56] Sachse, S., Rappert, A. & Galizia, C. G. The spatial representation of chemical structures in the antennal lobe of honeybees: steps towards the olfactory code. *Eur. J. Neurosci.* **11**, 3970–3982 (1999). 10583486 10.1046/j.1460-9568.1999.00826.x

[57] Pino, J. A., Marbot, R., Delgado, A., Zumárraga, C. & Sauri, E. Volatile constituents of propolis from honey bees and stingless bees from Yucatán. *J. Essent. Oil Res.* **18**, 53–56 (2006).

[58] Liendo, M. C. et al. Temporal changes in volatile profiles of Varroa destructor-infested brood may trigger hygienic behavior in Apis mellifera. *Entomol. Exp. Appl.* **169**, 563–574 (2021).

[59] Zhou, X. et al. Phylogenetic and transcriptomic analysis of chemosensory receptors in a pair of divergent ant species reveals sex-specific signatures of odor coding. *PLoS Genet.* **8**, e1002930 (2012). 22952454 10.1371/journal.pgen.1002930 PMC3431598

[60] Pask, G. M. et al. Specialized odorant receptors in social insects that detect cuticular hydrocarbon cues and candidate pheromones. *Nat. Commun.* **8**, 297 (2017). 28819196 10.1038/s41467-017-00099-1 PMC5561057

[61] McKenzie, S. K., Fetter-Pruneda, I., Ruta, V. & Kronauer, D. J. C. Transcriptomics and neuroanatomy of the clonal raider ant implicate an expanded clade of odorant receptors in chemical communication. *Proc. Natl. Acad. Sci. USA* **113**, 14091–14096 (2016). 27911792 10.1073/pnas.1610800113 PMC5150400

[62] Mondet, F. et al. Chemical detection triggers honey bee defense against a destructive parasitic threat. *Nat. Chem. Biol.* **17**, 524–530 (2021). 33495646 10.1038/s41589-020-00720-3

[63] Tórhalsdóttir, R. et al. Sensitivity to Cuticular Hydrocarbons Across the Odorant Receptor Family in the Indian Jumping Ant. 2025.07.17.665333 Preprint at 10.1101/2025.07.17.665333 (2025). 10.3389/finsc.2026.1666444 PMC13050904 41948125

[64] Esslen, J. & Kaissling, K.-E. Zahl und Verteilung antennaler Sensillen bei der Honigbiene (Apis mellifera L.). *Zoomorphologie* **83**, 227–251 (1976).

[65] de Bruyne, M. & Baker, T. C. Odor detection in insects: volatile codes. *J. Chem. Ecol.* **34**, 882–897 (2008). 18535862 10.1007/s10886-008-9485-4

[66] Akers, R. P. & Getz, W. A test of identified response classes among olfactory receptor neurons in the honeybee worker. *Chem. Senses* **17**, 191–209 (1992).

[67] Herre, M. et al. Non-canonical odor coding in the mosquito. *Cell* **185**, 3104–3123.e28 (2022). 35985288 10.1016/j.cell.2022.07.024 PMC9480278

[68] Badeke, E., Haverkamp, A., Hansson, B. S. & Sachse, S. A challenge for a male noctuid moth? Discerning the female sex pheromone against the background of plant volatiles. *Front. Physiol.* **7**, 143 (2016). 27199761 10.3389/fphys.2016.00143 PMC4843018

[69] Carcaud, J., Giurfa, M. & Sandoz, J.-C. Differential processing by two olfactory subsystems in the honeybee brain. *Neuroscience* **374**, 33–48 (2018). 29374539 10.1016/j.neuroscience.2018.01.029

[70] Deisig, N., Giurfa, M. & Sandoz, J. C. Antennal lobe processing increases separability of odor mixture representations in the honeybee. *J. Neurophysiol.* **103**, 2185–2194 (2010). 20181736 10.1152/jn.00342.2009

[71] Carcaud, J. et al. Multisite imaging of neural activity using a genetically encoded calcium sensor in the honey bee. *PLOS Biol* **21**, e3001984 (2023). 36719927 10.1371/journal.pbio.3001984 PMC9917304

[72] Chang, H. et al. *Non-Redundant Odorant Detection in a Locust*. http://biorxiv.org/lookup/doi/10.1101/2022.06.21.496967 (2022)

[73] Koutroumpa, F. A. et al. Heritable genome editing with CRISPR/Cas9 induces anosmia in a crop pest moth. *Sci. Rep.* **6**, 29620 (2016). 27403935 10.1038/srep29620 PMC4940732

[74] Liu, Q. et al. Deletion of the *Bombyx mori* odorant receptor co-receptor (*BmOrco*) impairs olfactory sensitivity in silkworms. *Insect Biochem. Mol. Biol.* **86**, 58–67 (2017). 28577927 10.1016/j.ibmb.2017.05.007

[75] Fandino, R. A. et al. Mutagenesis of odorant coreceptor Orco fully disrupts foraging but not oviposition behaviors in the hawkmoth Manduca sexta. *Proc. Natl. Acad. Sci.* **116**, 15677–15685 (2019). 31320583 10.1073/pnas.1902089116 PMC6681710

[76] Chen, Z. et al. Neurodevelopmental and transcriptomic effects of CRISPR/Cas9-induced somatic orco mutation in honey bees. *J. Neurogenet.* **0**, 1–13 (2021). 10.1080/01677063.2021.1887173 33666542

[77] Nouvian, M., Reinhard, J. & Giurfa, M. The defensive response of the honeybee Apis mellifera. *J. Exp. Biol.* **219**, 3505–3517 (2016). 27852760 10.1242/jeb.143016

[78] Brand, P. et al. The Nuclear and Mitochondrial Genomes of the Facultatively Eusocial Orchid Bee Euglossa dilemma. *G3 Genes Genomes Genet* **7**, 2891–2898 (2017). 10.1534/g3.117.043687 PMC5592917 28701376

[79] Karpe, S. D., Jain, R., Brockmann, A. & Sowdhamini, R. Identification of Complete Repertoire of Apis florea Odorant Receptors Reveals Complex Orthologous Relationships with Apis mellifera. *Genome Biol. Evol.* **8**, 2879–2895 (2016). 27540087 10.1093/gbe/evw202 PMC5630852

[80] Kelber, C., Rössler, W. & Kleineidam, C. J. Multiple olfactory receptor neurons and their axonal projections in the antennal lobe of the honeybee Apis mellifera. *J. Comp. Neurol.* **496**, 395–405 (2006). 16566001 10.1002/cne.20930

[81] Gonzalez, F., Witzgall, P. & Walker, W. B. I. Protocol for Heterologous Expression of Insect Odourant Receptors in Drosophila. *Front. Ecol. Evol*. **4**, (2016).

[82] Brand, P. et al. The evolution of sexual signaling is linked to odorant receptor tuning in perfume-collecting orchid bees. *Nat. Commun.* **11**, 244 (2020). 31932598 10.1038/s41467-019-14162-6 PMC6957680

[83] Chahda, J. S. et al. The molecular and cellular basis of olfactory response to tsetse fly attractants. *PLOS Genet* **15**, e1008005 (2019). 30875383 10.1371/journal.pgen.1008005 PMC6420007

[84] Chen, T.-W. et al. Ultrasensitive fluorescent proteins for imaging neuronal activity. *Nature* **499**, 295–300 (2013). 23868258 10.1038/nature12354 PMC3777791

[85] Lin, C.-C. & Potter, C. J. Re-Classification of Drosophila melanogaster Trichoid and Intermediate Sensilla Using Fluorescence-Guided Single Sensillum Recording. *PLOS ONE* **10**, e0139675 (2015). 26431203 10.1371/journal.pone.0139675 PMC4592000

[86] Marion-Poll, F. & Tobin, T. R. Software filter for detecting spikes superimposed on a fluctuating baseline. *J. Neurosci. Methods* **37**, 1–6 (1991). 2072733 10.1016/0165-0270(91)90015-r

[87] Pickett, J. A., Williams, I. H., Martin, A. P. & Smith, M. C. Nasonov pheromone of the honey bee,Apis mellifera L. (Hymenoptera: Apidae). *J. Chem. Ecol.* **6**, 425–434 (1980). 10.1007/BF00987702 24420593

[88] Ribbands, C. R. The scent perception of the honeybee. *Proc. R. Entomol. Soc. Lond. Ser. B Taxon.* **143**, 367–379 (1955).

[89] Boch, R., Shearer, D. A. & Shuel, R. W. Octanoic and Other Volatile Acids in the Mandibular Glands of the Honeybee and in Royal Jelly. *J. Apic. Res.* **18**, 250–252 (1979).

[90] Collins, A. M. & Blum, M. S. Alarm responses caused by newly identified compounds derived from the honeybee sting. *J. Chem. Ecol.* **9**, 57–65 (1983). 24408619 10.1007/BF00987770

[91] Bitterman, M. E., Menzel, R., Fietz, A. & Schäfer, S. Classical conditioning of proboscis extension in honeybees (Apis mellifera). *J. Comp. Psychol. Wash. DC 1983* **97**, 107–119 (1983). 6872507

[92] Pham-Delegue, M. H. et al. Behavioural discrimination of oilseed rape volatiles by the honeybee Apis mellifera L. *Chem. Senses* **18**, 483–494 (1993). 10.1093/chemse/22.4.391 9279462

[93] Laska, M., Galizia, C. G., Giurfa, M. & Menzel, R. Olfactory Discrimination Ability and Odor Structure–Activity Relationships in Honeybees. *Chem. Senses* **24**, 429–438 (1999). 10480679 10.1093/chemse/24.4.429

[94] Abramson, C. I., Wanderley, P. A., Wanderley, M. J. A., Silva, J. C. R. & Michaluk, L. M. The effect of essential oils of sweet fennel and pignut on mortality and learning in africanized honeybees (Apis mellifera L.) (Hymenoptera: Apidae). *Neotrop. Entomol.* **36**, 828–835 (2007). 18246255 10.1590/s1519-566x2007000600002

[95] Drezner-Levy, T. & Shafir, S. Parameters of variable reward distributions that affect risk sensitivity of honey bees. *J. Exp. Biol.* **210**, 269–277 (2007). 17210963 10.1242/jeb.02656

[96] Batish, D. R., Singh, H. P., Kohli, R. K. & Kaur, S. Eucalyptus essential oil as a natural pesticide. *For. Ecol. Manag.* **256**, 2166–2174 (2008).

[97] Swanson, H. L., Xinhua, Z. & Jerman, O. Working memory, short-term memory, and reading disabilities: a selective meta-analysis of the literature. *J. Learn. Disabil.* **42**, 260–287 (2009). 19255286 10.1177/0022219409331958

[98] Schiestl, F. P. The evolution of floral scent and insect chemical communication. *Ecol. Lett.* **13**, 643–656 (2010). 20337694 10.1111/j.1461-0248.2010.01451.x

[99] Reinhard, J., Sinclair, M., Srinivasan, M. V. & Claudianos, C. Honeybees Learn Odour Mixtures via a Selection of Key Odorants. *PLoS ONE* **5**, e9110 (2010). 20161714 10.1371/journal.pone.0009110 PMC2817008

[100] Suckling, D. M. & Sagar, R. L. Honeybees Apis mellifera can detect the scent of Mycobacterium tuberculosis. *Tuberculosis* **91**, 327–328 (2011). 21546308 10.1016/j.tube.2011.04.008

[101] Carroll, M. J. & Duehl, A. J. Collection of volatiles from honeybee larvae and adults enclosed on brood frames. *Apidologie* **43**, 715–730 (2012).

[102] Kaspi, R. & Shafir, S. Associative olfactory learning of the red dwarf honey bee Apis florea. *Apidologie* **44**, 100–109 (2013).

[103] Lawal, B. et al. Haematopoetic effect of methanol extract of Nigerian honey bee (Apis mellifera) propolis in mice. *J. Coast. Life Med.* **3**, 648–651 (2015).

[104] Paoli, M. et al. Differential Odour Coding of Isotopomers in the Honeybee Brain. *Sci. Rep*. **6**, (2016). 10.1038/srep21893 PMC4762004 26899989

[105] Wen, P. et al. Foragers of sympatric Asian honey bee species intercept competitor signals by avoiding benzyl acetate from Apis cerana alarm pheromone. *Sci. Rep.* **7**, 6721 (2017). 28751766 10.1038/s41598-017-03806-6 PMC5532208

[106] Annamma Abraham, A., Verghese, A. & Muthangi, S. Role of colour and volatile in foraging behaviour of honeybee Apis cerana on Jacquemontia pentanthos. *J. Asia-Pac. Entomol.* **21**, 1122–1128 (2018).

[107] Favaro, R., Roved, J., Haase, A. & Angeli, S. Impact of Chronic Exposure to Two Neonicotinoids on Honey Bee Antennal Responses to Flower Volatiles and Pheromonal Compounds. *Front. Insect Sci*. **2**, (2022). 10.3389/finsc.2022.821145 PMC10926470 38468759

[108] Gascue, F., Marachlian, E., Azcueta, M., Locatelli, F. F. & Klappenbach, M. Antennal movements can be used as behavioral readout of odor valence in honey bees. *IBRO Neurosci. Rep.* **12**, 323–332 (2022). 35746975 10.1016/j.ibneur.2022.04.005 PMC9210461

[109] Bates, D., Mächler, M., Bolker, B. & Walker, S. Fitting Linear Mixed-Effects Models Using lme4. *J. Stat. Softw.* **67**, 1–48 (2015).

[110] Bhandawat, V., Olsen, S. R., Gouwens, N. W., Schlief, M. L. & Wilson, R. I. Sensory processing in the Drosophila antennal lobe increases reliability and separability of ensemble odor representations. *Nat. Neurosci.* **10**, 1474–1482 (2007). 17922008 10.1038/nn1976 PMC2838615

[111] Vinje, W. E. & Gallant, J. L. Sparse coding and decorrelation in primary visual cortex during natural vision. *Science* **287**, 1273–1276 (2000). 10678835 10.1126/science.287.5456.1273

[112] Perez-Orive, J. et al. Oscillations and sparsening of odor representations in the mushroom body. *Science* **297**, 359–365 (2002). 12130775 10.1126/science.1070502

[113] Andreu, B. et al*.* Data from: Identification of two odorant receptors tuned to alarm pheromone in the honey bee Apis mellifera [Dataset]. Dryad. 10.5061/dryad.rv15dv4k2 (2025). 10.1038/s42003-025-09391-z PMC12848076 41436626

[114] Shearer, D. A. & Boch, R. 2-heptanone in the mandibular gland secretion of the honey-bee. *Nature* **206**, 530–530 (1965).5831852

[115] Winston, M. L. et al. The influence of queen mandibular pheromones on worker attraction to swarm clusters and inhibition of queen rearing in the honey bee (Apis mellifera L. *Insectes Sociaux* **36**, 15–27 (1989).

[116] Slessor, K. N., Kaminski, L.-A., King, G. G. S., Borden, J. H. & Winston, M. L. Semiochemical basis of the retinue response to queen honey bees. *Nature* **332**, 354–356 (1988).

[117] Free, J. B. *Pheromones of Social Bees*. (Comstock Pub. Associates, 1987).

[118] Keeling, C. I., Slessor, K. N., Higo, H. A. & Winston, M. L. New components of the honey bee (Apis mellifera L.) queen retinue pheromone. *Proc. Natl. Acad. Sci. USA* **100**, 4486–4491 (2003). 12676987 10.1073/pnas.0836984100 PMC153582

[119] Williams, I. H., Pickett, J. A. & Martin, A. P. The Nasonov pheromone of the honeybeeApis mellifera L. (Hymenoptera, Apidae). Part II. Bioassay of the components using foragers. *J. Chem. Ecol.* **7**, 225–237 (1981). 24420468 10.1007/BF00995745

[120] Boch, R. & Shearer, D. A. Identification of nerolic and geranic acids in the nassanoff pheromone of the honey bee. *Nature* **202**, 320–321 (1964).

[121] Le Conte, Y., Arnold, G., Trouiller, J., Masson, C. & Chappe, B. Identification of a brood pheromone in honeybees. *Naturwissenschaften* **77**, 334–336 (1990).

