# Supplementary material for: Identification of two odorant receptors tuned to alarm pheromone compounds in the honey bee Apis mellifera
Source: Commun Biol. 2025 Dec 23;9:115. doi: 10.1038/s42003-025-09391-z (PMC12848076; doi:10.1038/s42003-025-09391-z)
Supplement: Supplementary file 1 — Supplementary Material [file 42003_2025_9391_MOESM1_ESM.pdf]

Supplementary Material

Supplementary Table 1

	Primer	Reverse
*Amel*OR12	GAGAAGGTGGTGCAAGAGAAGA	GCCAATGAAGCAAGTGATCTGG
*Amel*OR71	CAAGATTTACGCCGTGTTCTCC	GTCAGCTTCTCGAACTTCAGGA
*Amel*OR83	TTACCCAGCGTACCCCATACTA	CGACTATCCTTCTCTCGGGTTTC
*Amel*OR109	CATCTACCCCACCTACTACTTCG	CCAGATACACGCTACTCAACACC
*Amel*OR115	GAATCGCCACAATCATCTGAGC	TGGTAGGTGCTCAGTCCAATTC
AmelOR136	CTATCGAGCTGGTGGACATCATG	GGGTGGTGAAGAAGATGAAGGAG
*Amel*OR160	CTGACCATTGGAGCTACAGGAG	TTCCACCAGAGCCTTCATCTTG
*Amel*OR163	GTGGATAATACCCGCCTGAGTAG	CGAATGGCAGCTTCATCTTCAG

Supplementary Figure 1

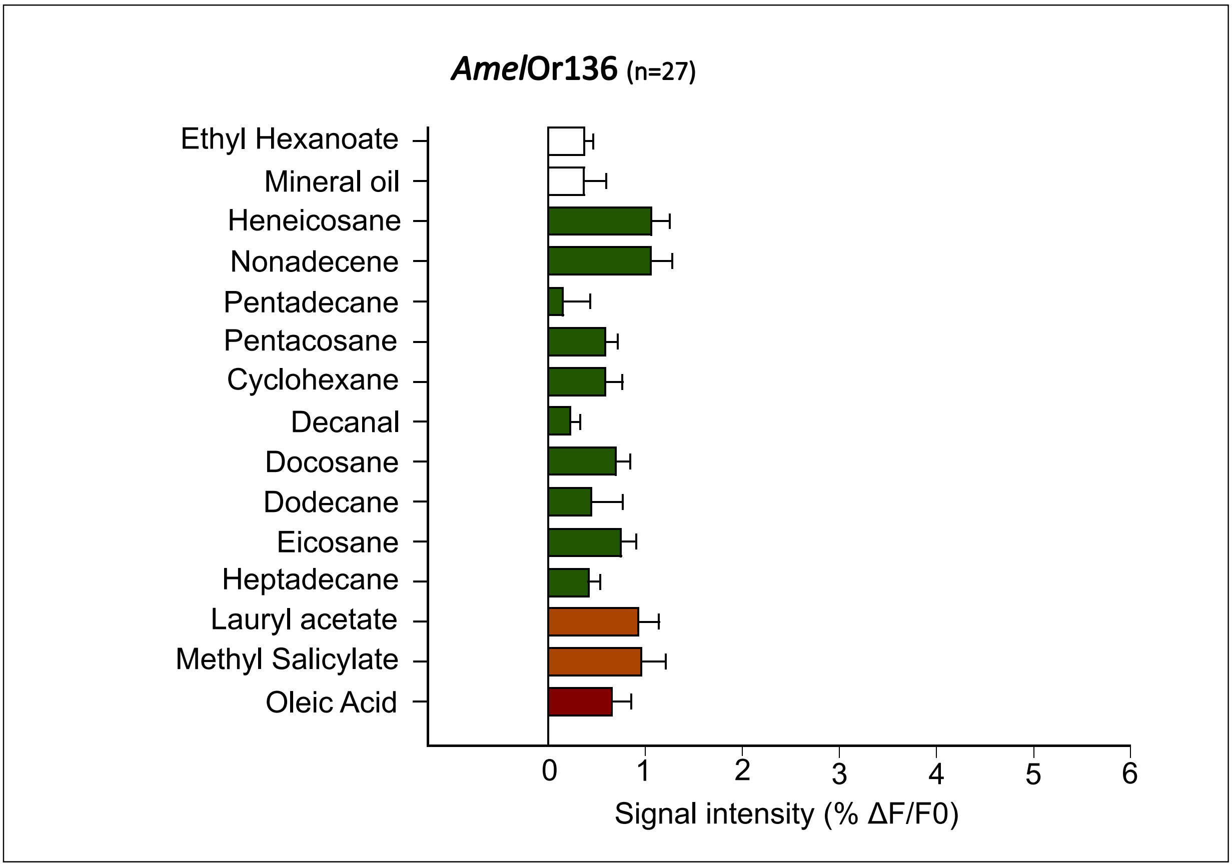

Calcium imaging responses (ΔF /F0) to compounds identified by GC-MS in worker honey bee extracts activating OSNs expressing AmelOR109 or AmelOR136. Control stimuli are represented in white, alkanes are represented in dark green, esters in orange and fatty acids in red. While significant heterogeneity in the responses was observed (Friedman test, Chi2=33.3, ddl=14, $p=0.002$), none of the comparisons with the mineral oil control were significant (Wilcoxon post-hoc tests, $p>0.052$).
