# Supplementary material for: Identification of two odorant receptors tuned to alarm pheromone compounds in the honey bee Apis mellifera
Source: Commun Biol. 2025 Dec 23;9:115. doi: 10.1038/s42003-025-09391-z (PMC12848076; doi:10.1038/s42003-025-09391-z)
Supplement: Supplementary file 2 — Description of Additional Supplementary Files [file 42003_2025_9391_MOESM2_ESM.docx]

**Description of Additional Supplementary File**

**File name**: Supplementary Data 1

**Description:** Sequences of the *AmelOR*
