# Supplementary material for: Identification of two odorant receptors tuned to alarm pheromone compounds in the honey bee Apis mellifera
Source: Commun Biol. 2025 Dec 23;9:115. doi: 10.1038/s42003-025-09391-z (PMC12848076; doi:10.1038/s42003-025-09391-z)
Supplement: Supplementary file 3 — Supplementary Data 1 [file 42003_2025_9391_MOESM3_ESM.pdf]

Supplementary Material

Supplementary Data 1

>*Amel*OR12

MIPLKFLSWPVGTWPFQVHEIFSISRTIFSISLLLLMVVILQVELYLDRSNAENNLDALL
MARIRNAKDGIRHTFWFAYPFSRMLGYWPLSVSSSAFAKISNYFIIFLSYLLTLIFMVPG
LLYIFLKVKNGRSRIKLLMSHINGIVQMAKYTILLRKTKEIAKLLDEIKKDWMTASEENR
QIFSTRASIEHKLTMVVVVTMYGGGFFYRAILPLSKGKIVLSNNVTIRLLPCPGYFGFLD
EQVSPNYEIIFTLQVLGGFVIYTAVCGTKSICLMLCLHMCGLLKILTNKVMELTNDKDEK
VVQEKIAHIVDYQTRIIEFLNDLNQFVPSVYFFEIILEVLIICIIGYCLITEWEDNNTMA
TVIFVIFQITCFIGTFAVCYAGQLLVDESENVRQACSTLNWYRLPVKKARSLILLILMSN
YPIKVTAGRIVDVSLVTFTSIIKNSVGYMNILQQVT

>*Amel*OR71

MRILRWTFLLFALCGCFPPSSWTTRLKRYLYKIYAVFSFVALNSFLLSQILDMV
YNVKGTDDFSDNFSVTVVVFVTCFKLITILTRRENILLLCNTLKQEPLSPINTE
EFEIFLKFEKLTDWNTLGYFILLMSSSLCILMGSLLANFKIRKLAFRTWLPYDY
STASAFLLAFAYQVVVATVCTFACVASDTLYSGLLIHISCQFEILEHRLKNIGS
DKNYTMKQCVRHHNHIYKYGEMVNDAFQSIMFFQFCTSLSMICFNFYRIMQIEM
DSRYVGTILYMVCSLMQIFYYCWFSNEVKLKSLELSDMIFRSNWTSLNNNVQRA
ILLVMRRSMKPIEFTSIYIVSVNLDSFMTLLKSSYSAFSVLQQSRES

>*Amel*OR83

MQTDNQLDISISLSTFFLKNVGVWMPDNSDEQRRMKMLFLYTIWMLFCGTIIST
RDLYFTLLYNGDILYAMTNTITTIMALIKICIILTYKGKFLNLIVYMQQNFWNV
DYDCQEKEILDDCRKTCIFFISSVTTIGMCTVMSYLTTPVITQSGSNESERMFP
FNIWINLPITRTPYYQIIFFVQGVSVYYIGISYFCFDNIFCIMAVHLAGQFRIL
RYRLMTLCDTEPETREKDSRSTFAKQVYKFYEQFKKCVRYHQALIDYYQNLENV
YTIITLGQVLVFSVLICLFGYQVFVAAASTARRFIFVFLLSGSMFLLFMFTYSC
NDVMEHSDNVAIGAYSALWTILPMDKFGRMLRNDLIMVIKRSRRVCYLTANGFF
PVSLETYTKILSTAVSYFTLLNNRVENAN

>*Amel*OR109

MDERAIEDQYLKINKFFGQLVGVWPYQKKFFKTCIRFITFTIMIFSLATQISRV
IVFYSLDVLSDQLPYINAGIVTLFKQYNYILNEDKLRELLHDIVSDRLIERSKE
ELEILEMYSRRTTALCALYKVMVYSCAFMFLVIPTIPPILNIVAPLNVSRSREF
IYPTYYFVDEQKYYYPILTHMIAVILVLSSVYLACDTNLVQIVHHGCALLAISG
YHFKHAVDDVKFCDGYYIDASMDETYVKIRQSIKAHKTAVQYVDKIDACHIHYF
LLVIGMIVLAFTGTFLKLSTMEVGIRFFTFCAYTIAQLIHLFFLTIMGQFLINA
NEETFKTIYEADWYNGSSKMQSLYVLVLRKCLSPPKLTGGGFVALNLDSFVQIL
KASFSYYTVFRS

>*Amel*OR115

MDFAMGWNRFNLTLLGVYPEPRKMSRNSRLMSSLIFWFTTLVTFTFICAPQTAN
LILKSTSLDEVIENLSINIPIVFALIKQIVLRYYKKALTELLGEMLADWSGPIG
DQDRETMLRNARLSRAISIVCSTLTYFMLLAFVSLQVWSNAENASETDLGGLLH
PATFPYETSKSPNYEITWLGQLMGTVLTAICYSCFDTFLAVLVLHLCGQLTVLG
TALEDLVNATRRNDYKTFEQRLSSIVNRHNHLSRFAVIVEDCFNITLLVQTLIC
TAMFCLTGYRMITSVDREDEADVPIVGIIFFIIHVIYTMLHLFIYCYVGETLLG
QSTGIGLSTYHCNWYDLPSRRAVLLMIVIRRANVSFQITAGKFSPFSLEFFNAV
LKTSAGYLSVLLAMKDRLVEGK

>*Amel*OR136

MNVFDKHYHTYRTLMKIVGLWPYNNSIYVWIQRLWFLMFFFGNIIFQIMSLLTS
AITLQNCVLIFSTTCPLIIVLFRYIGLILFFPTIKLLFHHMCMEEAMIQDSIEA
QIRRKYIDDSCYMIDIFFWMTYVGIALCSILLLCPITLDFIMPLNESRTRIVHY
VTIFSDKSIIYMDILCLNYMLLAILVILSATCTESILGLYSYHTSIMFKIIGHR
IQKIVKYLTMFNLSSKQIDSKLAELYRIVDIHNQAIELVDIMINNSGKQFMIST
LLSVISMAISLHRLLNAIVIKKDELEILISFIFFTTQLVITFLNNNCNQILIDN
SQELFIELYISMWYFVPLKVQKILLLIMIRSSTACMINILGVFTPCYIGFSKML
STSFSYFTLMHSIQ

>*Amel*OR160

MRRPISSYVELFYDKNVISWSKRLLGLSGLWPDNRNDVRFFLYITYVVIFTWLE
IVTLVQNIHDLEKTLKNITLSFPTILIVLKAVMFRMNMHLVLPLLTVVKRDVNE
GLYRSAEERRTVVWYNVAATLFSTSSALSLFFVPTLFYAKPIIGCLLSKYNNCT
LPFELPMKVNNVYEITKLQTYALFCVYLIPTSTLLTIGATGADSLLVTLTFHLC
SQLSIVAYRMRNVNIEPKIYFPKMKALVERHTELLRLANILANTFSSLMFVQTL
GLIFSLCIVVYQLLMTSESGEDMNTIHFIIYSCAVILLAFCYCFLGECLINESS
EVQMACYFTNWYDLPEQYTRSLIFCIARAQKPLYLTAGKFYVFSLETFAVIVKA
SMAYLSVLKSII

>*Amel*OR163

MFKTIITYPVEVCLRLIGVWPYSSYRIMQRIFWTIIMGNSTVFQLWYCISYFKT
ADLFDLLDGITLTLSNTVTFFKLIILWFNYRTIHNILTIVFEDWNNRALTDKKK
QLMVDNTRLSSRISNFLFGIYSVTCILYSASIALISDDIDNTNNELILNNKKLL
LKMKLPFDFTIFPLYEFVIVAQFVFECFVALTAGMLMAFSAALVLHIGSQIDIT
CQELIEIPRHKGKTSYILKNIIVKHQRILRLSENVKYLFLYTSLIQFLSNILVI
CFLGFILVNALGTEQESTIFIKCFPYYIAANCEAFILCYTGEYLMFKNESIVHA
AYDTLWYNLNPRDSRIVLLILIQAQRKLILSAGNFVTLSVQTFASMQKVSASYI
SILMTIY
